# Lamin chromatin binding is modulated by interactions of different LAP2α domains with lamins and chromatin

**DOI:** 10.1016/j.isci.2024.110869

**Published:** 2024-09-02

**Authors:** Daria Filipczak, Anna Souchet, Konstantina Georgiou, Roland Foisner, Nana Naetar

**Affiliations:** 1 Max Perutz Labs, Vienna Biocenter Campus (VBC), Dr.-Bohr-Gasse 9 / Vienna Biocenter 5, Vienna 1030, Austria; 2 Medical University of Vienna, Max Perutz Labs, Dr.-Bohr-Gasse 9 / Vienna Biocenter 5, Vienna 1030, Austria; 3 Vienna BioCenter PhD Program, a Doctoral School of the University of Vienna and the Medical University of Vienna, Vienna A-1030, Austria

**Keywords:** Nucleic acids, Chromosome organization, Molecular interaction, Cell biology

## Abstract

Lamins A and C are components of the lamina at the nuclear periphery and associate with heterochromatin. A distinct, relatively mobile pool of lamin A/C in the nuclear interior associates with euchromatic regions and with lamin-associated polypeptide 2α (LAP2α). Here we show that phosphorylation-dependent impairment of lamin assembly had no effect on its chromatin association, while LAP2α depletion was sufficient to increase chromatin association of lamins. This suggests that complex interactions between LAP2α, chromatin, and lamins regulate lamin chromatin binding. Both the C terminus of LAP2α and its N-terminal LAP2-Emerin-MAN1 (LEM) domain, mediating interaction with lamin A/C indirectly via barrier-to-autointegration factor (BAF), are required for binding to lamins. The N-terminal LEM-like domain of LAP2α, but not its LEM domain, mediates chromatin association of LAP2α and requires LAP2α dimerization via its C terminus. Our data suggest that formation of several LAP2α-, lamin A/C-, and BAF-containing complexes in the nucleoplasm and on chromatin affects lamin chromatin association.

## Highlights

• LAP2α, lamin A/C, and BAF form different soluble and chromatin-bound complexes
• Various LAP2α domains cooperatively contribute to lamin A/C and chromatin binding
• LAP2α complexes compete with lamin complexes for chromatin association
• LAP2α binding to lamin A/C in the nucleoplasm restricts lamin chromatin binding

## Graphical abstract

**Figure:**
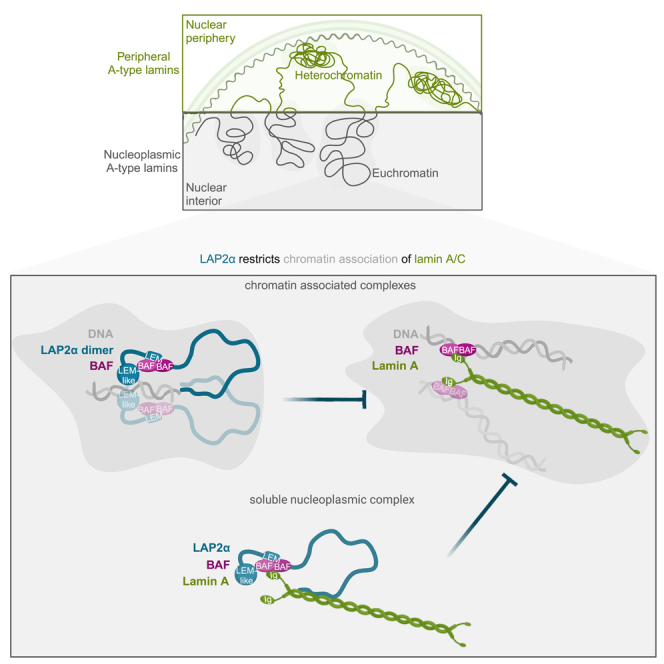

## Introduction

The nuclear lamina is a scaffold structure underneath the inner nuclear membrane, primarily composed of lamins and associated proteins.^1^ It is a key element for the spatial organization of chromatin, as it anchors heterochromatic genomic regions, known as lamina-associated domains (LADs), to the nuclear periphery, and is involved in cell-type specific gene repression.^2^^,^^3^^,^^4^ Lamins are intermediate filament-type proteins classified into A- and B-type lamins. B-type lamins are ubiquitously expressed, whereas A-type lamin expression is developmentally regulated, with low levels in embryonic stem cells and upregulation during differentiation.^5^ B-type lamins are post-translationally processed by sequential reactions, including farnesylation at a cysteine residue in their C-terminal CaaX sequence (C = cysteine, a = aliphatic, and X any amino acids), proteolytic cleavage of the three C-terminal residues, and carboxymethylation at the farnesylated cysteine.^6^ These hydrophobic modifications mediate strong interaction of B-type lamins with the inner nuclear membrane.^7^ The A-type lamin, lamin A, is initially modified similarly as lamin B but undergoes an additional step of post-translational processing, leading to the removal of its last 15 amino acids, including the farnesylated and carboxymethylated cysteine.^8^ Lamin C, a smaller isoform of lamin A, lacks a CaaX motif and is never farnesylated and carboxymethylated. Consequently, lamins A and C are less tightly bound to the inner nuclear membrane and a fraction of A-type lamins is found throughout the nucleus in a relatively dynamic pool.^5^^,^^9^ While the molecular structure and functions of lamin filaments at the nuclear periphery are well understood, very little is known about the structure, assembly state, regulation, and functions of the intranuclear lamin A/C pool.^9^^,^^10^

Lamins at the nuclear periphery form 3.5 nm wide filaments^1^^,^^11^ and interact with numerous inner nuclear membrane proteins.^12^^,^^13^^,^^14^ Nucleoplasmic lamin A/C interacts with lamin-associated polypeptide 2 alpha (LAP2α), a splice variant of the *Lap2* gene (*TMPO*), which encodes up to six isoforms.^15^^,^^16^ Whereas most LAP2 isoforms are integral proteins of the inner nuclear membrane and interact with lamin filaments at the periphery, LAP2α lacks a transmembrane domain and localizes throughout the nucleoplasm. All LAP2 isoforms share the N terminus, which includes a LAP2-Emerin-MAN1 (LEM) domain, mediating interaction with the chromatin-binding protein barrier-to-autointegration factor (BAF), and a LEM-like motif reported to interact with DNA directly.^17^^,^^18^^,^^19^^,^^20^ Unlike the other LAP2 isoforms, LAP2α influences the mobility, assembly state, and nuclear localization of lamin A.^10^^,^^15^^,^^21^^,^^22^^,^^23^ Depletion of LAP2α leads to reduced mobility and solubility of lamin A/C in high-salt buffers and to the formation of higher-order lamin A/C structures that seem to stably interact with chromatin.^10^ Based on these studies, it was concluded that LAP2α maintains a relatively mobile pool of lamin A/C of an undefined assembly state in the nuclear interior, but the mechanisms remain elusive. Besides the interaction with LAP2α, phosphorylation of A-type lamins also affects their intranuclear localization and assembly state. In particular, phosphorylation of lamin A/C at serines 22 and 392,^24^ known to induce lamin filament disassembly at the onset of mitosis,^25^ was found to also regulate lamin localization during interphase.^26^ Lamin A containing phosphomimetic Ser-to-Asp mutations at ser22/392 mostly localizes in the nucleoplasm, while Ser-to-Ala phosphomutants are exclusively found at the nuclear periphery.

Both lamin A/C phosphorylation and interaction with LAP2α seem to affect lamin A/C chromatin interaction. Peripheral lamins predominantly interact with heterochromatic LADs, together with numerous inner nuclear membrane proteins, such as LEM domain proteins^27^^,^^28^^,^^29^^,^^30^ and lamin B receptor (LBR).^31^^,^^32^ In contrast, ser22-phosphorylated lamin A/C was found to interact with active enhancers in euchromatin.^33^ Nucleoplasmic A-type lamins and LAP2α associate with overlapping heterochromatic and euchromatic regions, most likely in the nuclear interior.^15^^,^^21^ Depletion of LAP2α in fibroblasts leads to rearrangement of lamin A/C on chromatin and to genome-wide epigenetic changes,^21^ but the molecular mechanisms involved remain elusive.

In this study, we systematically investigate how specific functional domains of LAP2α regulate association of A-type lamins with hetero- and euchromatin. We show that LAP2α weakens association of lamin A/C with chromatin, independent of lamin A/C assembly state and stable binding of LAP2α to lamins. Additionally, we demonstrate that LAP2α requires both its N-terminal LEM domain and its C-terminal domain to stably bind to lamin A/C, with the LEM domain mediating lamin A/C interaction indirectly via BAF. Surprisingly, whereas the BAF-interacting LEM domain is dispensable for LAP2α chromatin interaction *in vivo*, the LEM-like motif, as well as the dimerization of LAP2α mediated by its C terminus, significantly contributes to its association with chromatin. Overall, our data point toward a competitive association of LAP2α- and lamin A/C-containing complexes with overlapping genomic regions and shed light on the complex interactions between LAP2α, lamin A/C, and chromatin.

## Results

### LAP2α is sufficient to weaken lamin A/C association with hetero- and euchromatin, independent of its assembly state and formation of stable protein complexes

**Figure 1:**
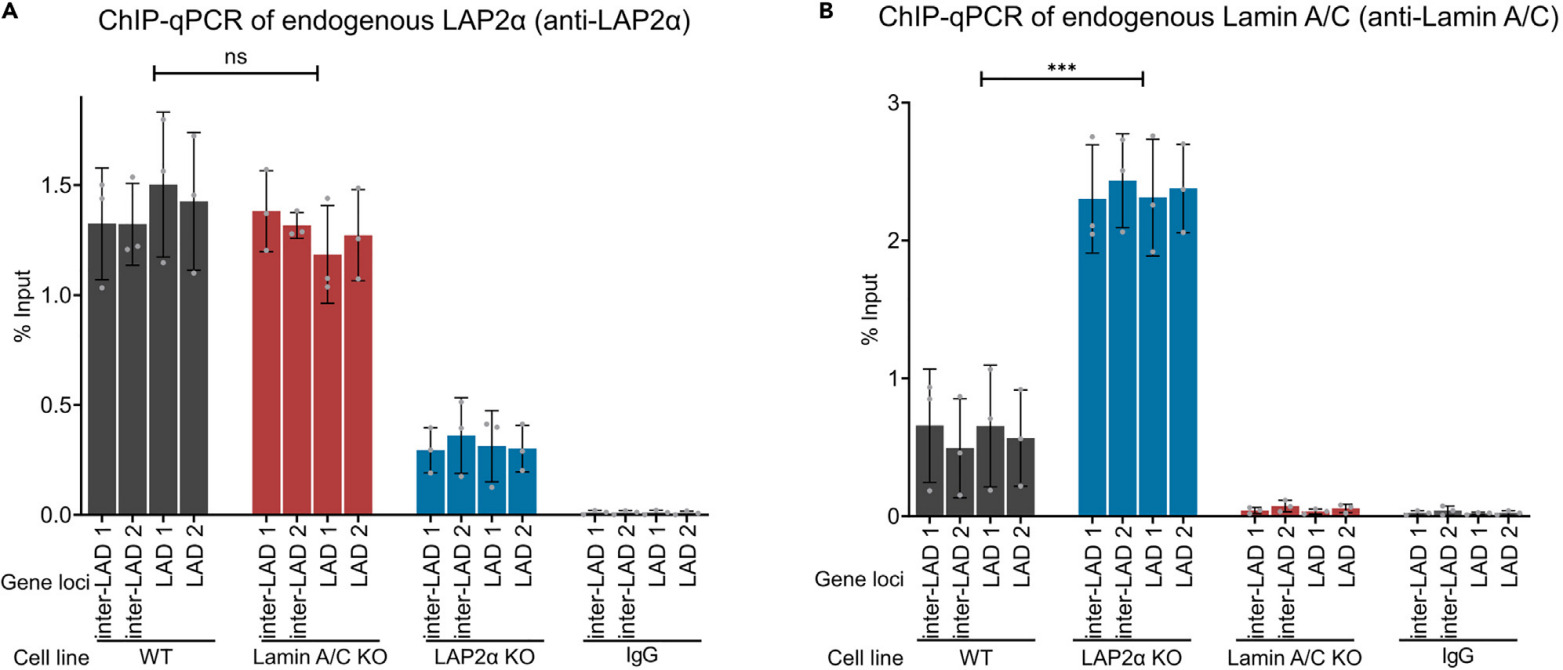
LAP2α is sufficient to weaken chromatin association of lamin A/C, but not vice versa (A and B) Probing chromatin interaction of LAP2α (A) and lamin A/C (B) by ChIP-qPCR. ChIP was performed using anti-LAP2α (A) or lamin A/C E1 (B) antibody and binding to LAP2α and lamin A/C-enriched genomic regions, previously identified by ChIP-seq^21^ (see also Figure S1) was probed by qPCR. Graphs display averages ±standard deviation from biological replicates. Percentage input was calculated and normalized between replicates to account for batch-to-batch variability. Paired t test, ∗∗∗*p* = 0.0001, non-significant (ns) *p* = 0.2981, n _all tested genotypes_ = 3; t = 1.256, degrees of freedom (df) = 3 (A), t = 25.18, df = 3 (B).

Previous genome-wide studies demonstrated that the patterns of lamin A/C association with hetero- and euchromatin change upon LAP2α knockout (KO) in fibroblasts compared to wild-type cells.^21^ To explore the interplay between the associations of LAP2α and lamin A/C with chromatin at a biochemical level, we first tested chromatin association of both proteins in wild-type versus the respective KO cells by chromatin immunoprecipitation (ChIP), followed by quantitative PCR (qPCR) analyses of selected genomic loci, previously identified to interact with these proteins^21^ (Figure S1). LAP2α efficiently associated with two loci within LADs (heterochromatin) and two loci outside LADs (euchromatin) (Figures 1A and S1, LAD versus inter-LAD), and chromatin binding remained unchanged upon lamin A/C depletion (Figure 1A). In contrast, A-type lamin ChIP revealed a significantly stronger lamin A/C signal on these hetero- and euchromatic genomic loci in the absence of LAP2α compared to the wild-type control (Figure 1B). These findings suggest a stronger and/or more stable association of lamin A/C with chromatin in the absence of LAP2α and imply that LAP2α weakens the binding of lamin A/C to chromatin.

**Figure 2:**
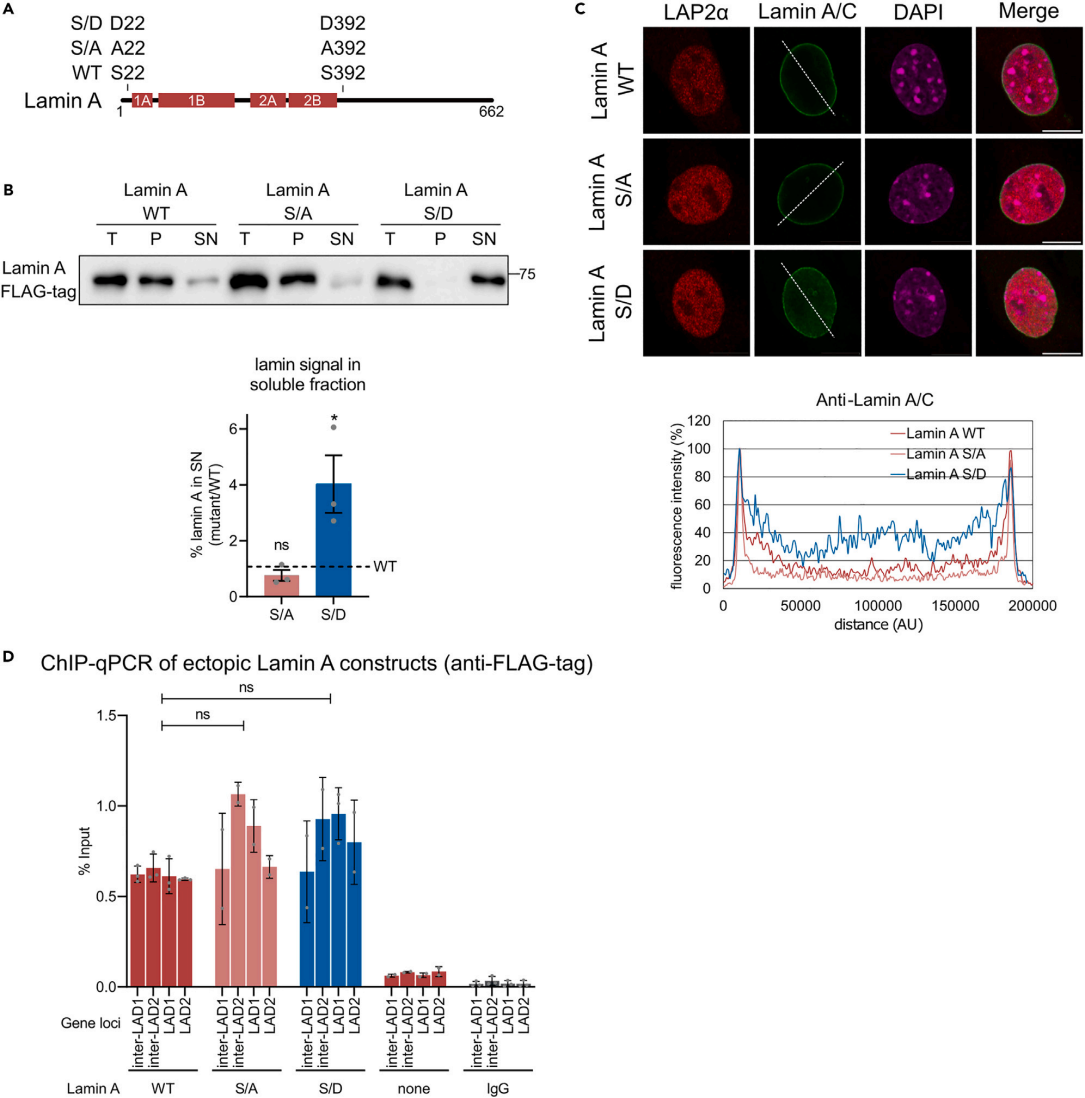
Assembly state of lamin A does not influence its interaction with chromatin (A) Schematic representation of lamin A constructs, ectopically expressed in a lamin A/C KO cell line. All constructs carry an FLAG tag at their N terminus. The graph highlights the positions of serines (S) 22 and 392, which represent major phosphorylation sites in lamin A/C. In the S/A variant of lamin A, serines were substituted with alanine (A), in the S/D variant with aspartic acid (D). 1A, 1B, 2A, and 2B are coil domains within α-helical rod of lamin A. (B) Solubility assays were conducted for wild-type (WT), S/A, and S/D variants of lamin A (see STAR methods for details). Upper panel: representative western blot of protein extracts using indicated antibody. T, total; P, pellet; SN, supernatant. Lower panel: quantification of western blot results. The graph represents the percentage of signal in the supernatant relative to the total fraction for S/A and S/D variants of lamin A, expressed as the average fold difference ±SEM compared to wild-type samples. Repeated-measures one-way ANOVA test, ns p_WT vs S/A_ = 0.9341, ∗p_WT vs S/D_ = 0.0320, n _all tested genotypes_ = 3 biological replicates; F (2, 4) = 10.56. (C) Lamin A/C KO cell line ectopically expressing WT, S/A, and S/D lamin A constructs was processed for immunofluorescence microscopy using anti-FLAG antibody detecting ectopic lamin A, 245.2 antibody targeting LAP2α, and DAPI for DNA visualization. Scale bar is 10 μm. Fluorescence intensity was assessed for each cell nucleus along the indicated dashed line in the green channel, and the results are presented as a percentage of the maximum value in the graphs below. (D) Probing chromatin interaction of ectopically expressed WT, S/A, and S/D lamin A constructs by ChIP-qPCR. ChIP was performed as described in Figure 1, using anti-FLAG antibody. Graphs display averages ±standard deviation from biological replicates. Repeated-measures one-way ANOVA test, non-significant (ns) p_WT vs S/A_ = 0.2182, p_WT vs S/D =_ 0.1167, n _all tested genotypes_ = 3, F (1.361, 4.084) = 69.19.

Based on previous studies showing that LAP2α keeps lamin A/C in a mobile, lower assembly state,^10^ we reasoned that LAP2α may reduce the association of lamin A/C with chromatin indirectly via impairing lamin filament assembly. To test whether the assembly state of lamin A influences its binding to chromatin, we ectopically expressed wild-type or an assembly-deficient mutant of lamin A in lamin A/C KO cells. As phosphorylation of lamin A/C on residues S22 and S392 has been shown to inhibit lamin A/C assembly into the lamina,^26^ we generated phosphomimetic S/D and non-phosphorylated S/A lamin A mutants and expressed these in lamin A/C KO cells (Figure 2A). As expected, the assembly-deficient phosphomimetic lamin A mutant (S/D lamin A) was nearly completely soluble in a buffer containing 0.5% detergent and 150 mM salt, whereas the majority of the ectopic wild-type lamin A and the S/A lamin A mutant were mostly retained in the insoluble pellet fraction (Figure 2B). Accordingly, immunofluorescence staining revealed that wild-type lamin A and the S/A lamin A mutant predominantly localized at the nuclear periphery, while the assembly-deficient S/D lamin A displayed a more homogeneous distribution across the nucleoplasm (Figure 2C). Line profiles of immunofluorescence intensity across the nucleus corroborated these localization patterns (Figure 2C, lower panel). Together, these results confirm that the phosphomimetic lamin A mutant exhibits an impaired assembly into the lamina scaffold and partially localizes to the nuclear interior. Despite these different biochemical properties of lamin mutants, their association with both hetero- and euchromatin was unaltered (Figure 2D). Thus, the assembly state of A-type lamins does not affect their association with chromatin.

**Figure 3:**
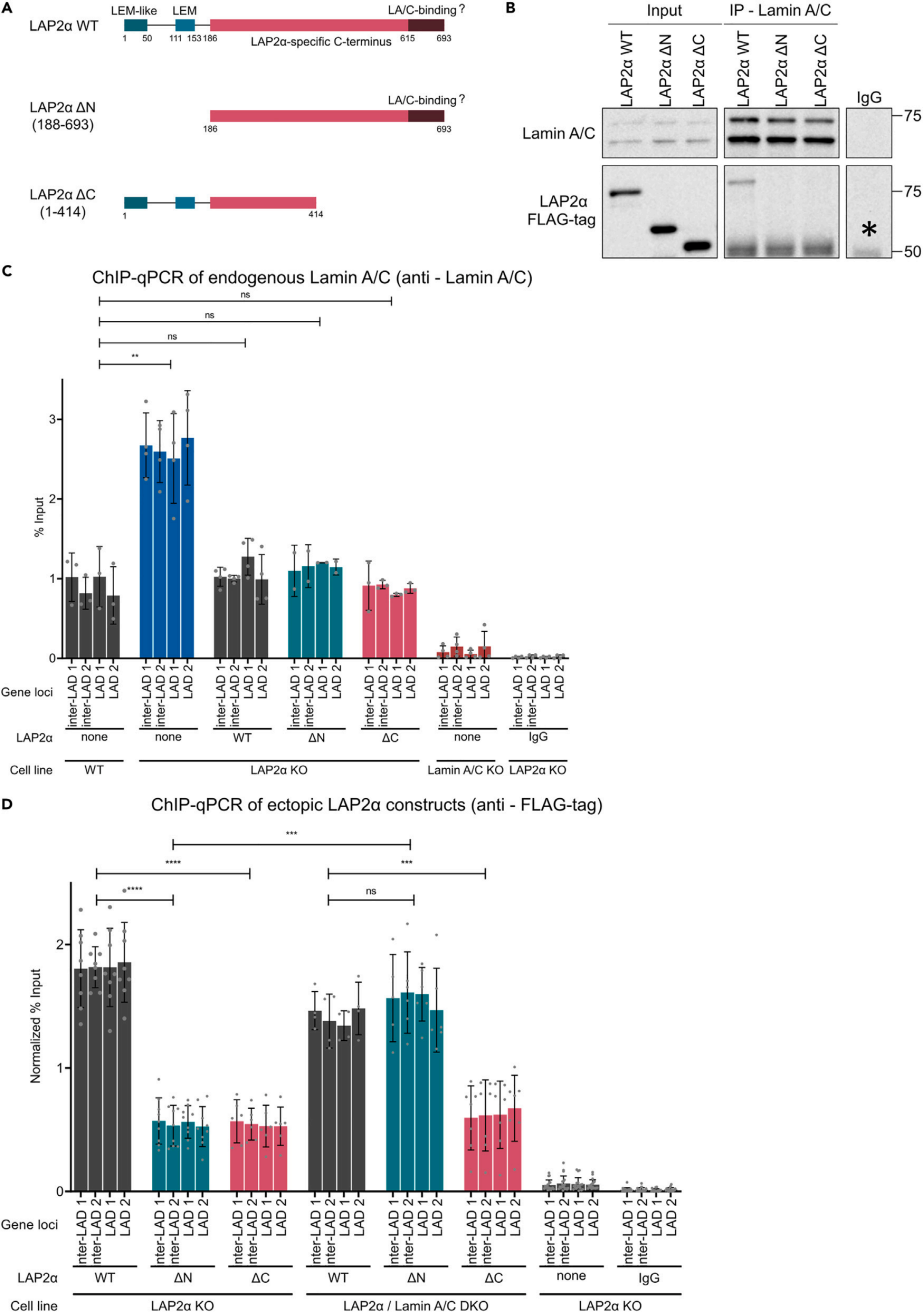
Inhibition of lamin A/C chromatin association by LAP2α does not require formation of stable protein complexes (A) Schematic representation of LAP2α and its deletion mutants. All constructs carry a FLAG tag at their N terminus. The LEM-like motif (residues 1–50) and the LEM domain (residues 111–152) in the N terminus of LAP2α are indicated. The C terminus harbors a previously identified lamin A/C-binding site (residues 615–693).^15^ The LAP2α ΔN mutant consists of residues 186–693, and the LAP2α ΔC mutant consists of residues 1–414. (B) Lamin A/C was immunoprecipitated from LAP2α KO cell lines ectopically expressing LAP2α WT, LAP2α ΔN, or LAP2α ΔC using the E1 anti-lamin A/C antibody. The immunoprecipitated samples were subsequently subjected to western blot analysis using the indicated antibodies. Asterisk denotes unspecific band. (C) Probing chromatin interaction of lamin A/C in WT, LAP2α KO cells, and LAP2α KO cells ectopically expressing wild-type LAP2α and its deletion mutants by ChIP-qPCR. ChIP was performed as described in Figure 1, using E1 anti-lamin A/C antibody. Graphs display averages ±standard deviation from biological replicates. Repeated-measures one-way ANOVA test, ∗∗p_none (WT) vs none (LAP2α KO)_ = 0.0015, ns p_none (WT) vs WT (LAP2α KO)_ = 0.1721, p_none (WT) vs ΔN (LAP2α KO)_ = 0.1125, p_none (WT) vs ΔC (LAP2α KO)_ = 0.9930, n _none (WT)_ = 3, n _none (LAP2α KO)_ = 4, n _WT (LAP2α KO)_ = 4, n _ΔN (LAP2α KO)_ = 3, n _ΔC (LAP2α KO)_ = 3, n _none (Lamin A/C KO)_ = 4, n _IgG (LAP2α KO)_ = 4; F (1.637, 4.912) = 337.9. (D) Probing chromatin interaction of ectopically expressed LAP2α and its deletion mutants in LAP2α KO and LAP2α/lamin A/C DKO cells by ChIP-qPCR. ChIP was performed as described in Figure 1, using anti-FLAG antibody. Graphs display averages ±standard deviation from biological replicates. Repeated-measures one-way ANOVA test, ∗∗∗∗p_WT (LAP2α KO) vs ΔN (LAP2α KO)_ < 0.0001, ∗∗∗∗p_WT (LAP2α KO) vs ΔC (LAP2α KO)_ < 0.0001, ns p_WT (LAP2α/ Lamin A/C DKO) vs ΔN (LAP2α/ Lamin A/C DKO)_ = 0.4863, ∗∗∗p_WT (LAP2α/ Lamin A/C DKO) vs ΔC (LAP2α/ Lamin A/C DKO)_ = 0.0008, ∗∗∗p _ΔN (LAP2α KO) vs ΔN (LAP2α/ Lamin A/C DKO)_ = 0.0003, n _WT (LAP2α KO)_ = 6, n _ΔN (LAP2α KO)_ = 9, n _ΔC (LAP2α KO)_ = 6, n _WT (LAP2α/ Lamin A/C DKO)_ = 4, n _ΔN (LAP2α/ Lamin A/C DKO)_ = 6, n _ΔC (LAP2α/ Lamin A/C DKO)_ = 7, n _none (LAP2α KO)_ = 19, n _IgG (LAP2α KO)_ = 19; F (1.537, 4.612) = 1221.

Next, we tested whether the LAP2α-mediated effect on the association of lamin A/C with chromatin requires the formation of stable LAP2α-lamin A/C complexes. LAP2α contains an N-terminal domain, comprising two proposed chromatin-binding regions: the LEM domain, a 40-amino-acid bi-helical motif binding to the DNA crosslinker protein BAF,^18^^,^^19^^,^^20^ and an LEM-like motif proposed to directly bind DNA.^18^^,^^19^ The C-terminal domain of LAP2α includes a region at the very C terminus, which contains potential lamin A/C interaction sites and which was shown to be sufficient to bind lamin C *in vitro*^15^^,^^34^ (Figure 3A). We expressed truncation mutants of LAP2α, missing either the N-terminal chromatin interaction domains (ΔN) or the C-terminal lamin-interaction domain (ΔC) in LAP2α KO cells (Figure 3A) and assessed stable interaction of LAP2α mutants with endogenous lamin A/C by co-immunoprecipitation using antibodies against lamin A/C. Intriguingly, while lamin A/C pulled down full-length LAP2α, it interacted with neither N- nor C-terminal truncation mutants (Figure 3B), indicating that stable interaction of these proteins *in vivo* is more complex than previously thought and may require the cooperation of various domains. Despite the lack of stable interactions of LAP2α truncation mutants with lamin A/C, re-expression of these mutants in LAP2α KO cells reduced the association of lamin A/C with chromatin to levels seen in wild-type cells, similar to the re-expression of ectopic full-length LAP2α (Figure 3C). These data indicate that the ability of LAP2α to weaken the association of lamin A/C with chromatin does not require formation of stable LAP2α-lamin A/C protein complexes.

### Both the N- and C-terminal domains of LAP2α contribute to efficient chromatin association, but only its C-terminal domain competes with lamin A/C for chromatin binding

To mechanistically understand the interplay between association of LAP2α and lamin A/C with chromatin, we first assessed the functions of various LAP2α domains in chromatin association. We ectopically expressed FLAG-tagged LAP2α fragments or mutants in LAP2α KO cells and performed ChIP of LAP2α.

As different LAP2α mutants may be expressed at different levels in the nucleus, we first tested if and how nuclear protein levels affect ChIP signals. We compared the ChIP signal obtained for endogenous LAP2α, which is expressed at low levels, with that of ectopic full-length LAP2α, expressed at much higher levels, and found a positive correlation between protein levels and ChIP signals (Figures S2A and S2B). However, when we normalized the obtained ChIP signals to the protein levels determined by densitometric analysis of immunoblots, we observed similar efficiencies of chromatin association for both endogenous and ectopic LAP2α (Figure S2C). Additionally, immunofluorescence microscopy and immunoblot analyses of cytoplasmic and nuclear fractions showed that, unlike wild-type LAP2α and LAP2α ΔN mutant, which localized exclusively to the nucleus, other mutants such as the LAP2α ΔC mutant were found in both the nucleoplasm and the cytoplasm (Figures S3 and S4). To account for the differences in cellular localization and expression levels of LAP2α fragments in the nucleus, we measured the relative nuclear protein levels of all LAP2α fragments by densitometric analysis of protein bands in immunoblots of nuclear fractions. These measurements were then used to normalize the corresponding LAP2α ChIP signals (Figure S4).

Using this normalization approach, we first compared the chromatin association of full-length LAP2α and its truncation variants, LAP2α ΔN and ΔC, expressed in LAP2α KO cells, by ChIP analyses with antibodies targeting the FLAG tag. Interestingly, the normalized chromatin association of both LAP2α ΔN and ΔC was significantly lower than that of full-length protein (Figure 3D, LAP2α KO cells), indicating that both the N- and C-terminal domains of LAP2α contribute to its association with hetero- and euchromatin *in vivo*. Strikingly, in the absence of endogenous lamin A/C (LAP2α/lamin A/C double knockout [DKO] cells), LAP2α ΔN bound to chromatin with the same efficiency as full-length LAP2α. This was in stark contrast to its reduced chromatin association in cells expressing lamin A/C (Figure 3D, compare LAP2α KO versus LAP2α/lamin A/C DKO cells). However, the chromatin association of the LAP2α ΔC mutant remained consistently low, regardless of the presence or absence of lamin A/C.

Overall, these results show that both the N- and C-terminal domains of LAP2α contribute equally to efficient binding to chromatin in the presence of lamin A/C. Conversely, in the absence of lamin A/C, the C terminus of LAP2α (LAP2α ΔN) interacts with chromatin as strongly as full-length LAP2α. As LAP2α ΔN does not stably bind lamin A/C *in vivo* (Figure 3B), we favor a model where LAP2α ΔN and lamin A/C compete for chromatin-binding sites. In contrast, LAP2α ΔC exhibits less robust chromatin binding in the presence and absence of lamin A/C, probably due to its generally weaker association with chromatin compared to LAP2α ΔN.

### The LEM-like domain of LAP2α is the main site for chromatin association, while its LEM domain mediates stable interaction with lamin A/C via BAF

**Figure 4:**
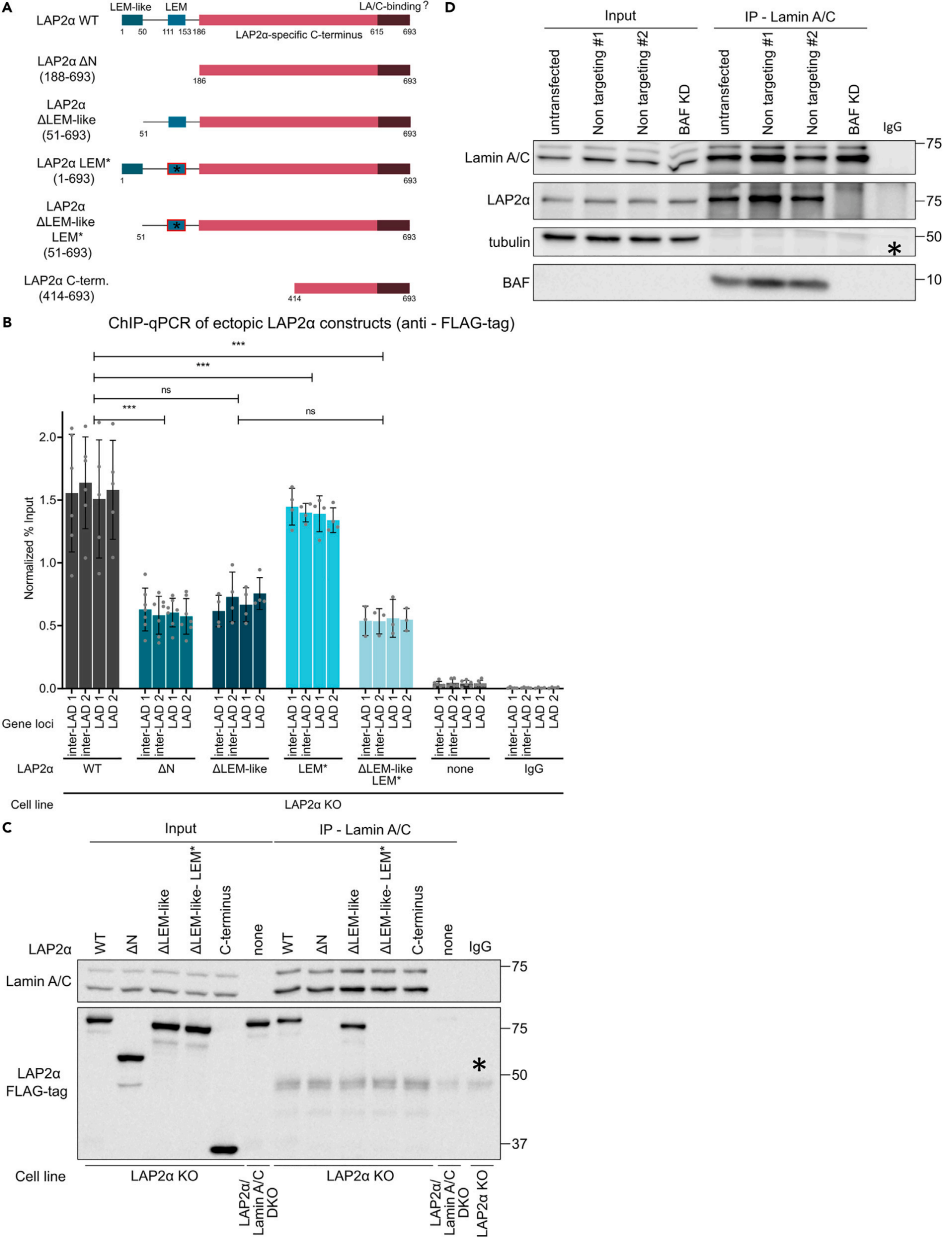
Binding of the LEM domain of LAP2α to BAF is required for LAP2α-lamin A/C interaction (A) Schematic representation of LAP2α and its deletion mutants. All constructs carry a FLAG tag at their N terminus. The LAP2α ΔN mutant is as described in Figure 3A. The LAP2α ΔLEM-like mutant consists of residues 51–693 and lacks the LEM-like DNA-interaction site. The LAP2α LEM∗ mutant harbors 4-point mutations (residues 130–133) in its LEM domain, disabling its binding to BAF.^35^ The LAP2α ΔLEM-like-LEM∗ mutant lacks the LEM-like DNA-interaction site and harbors 4-point mutations (residues 130–133) in its LEM domain. The LAP2α C-terminal mutant (LAP2α C-term.) consists of residues 414–693, corresponding to the very C-terminal part of LAP2α. (B) Probing chromatin interaction of ectopically expressed LAP2α and its deletion mutants in LAP2α KO cells by ChIP-qPCR. ChIP was performed as described in Figure 1, using anti-FLAG antibody. Graphs display averages ±standard deviation from biological replicates. Repeated-measures one-way ANOVA test, ∗∗∗p_WT vs ΔN_ = 0.0005, ∗∗∗p_WT vs ΔLEM-like_ = 0.0003, ns p_WT vs LEM∗_ = 0.0816, ∗∗∗p_WT vs ΔLEM-like-LEM∗_ = 0.0003, n _WT_ = 6, n _ΔN_ = 7, n _ΔLEM-like_ = 4, n _LEM∗_= 4, n _ΔLEM-like-LEM∗_= 3, n _none_ = 6, n _IgG_ = 6; F (1.552, 4.657) = 997.1. (C and D) Lamin A/C was immunoprecipitated from LAP2α KO cell lines ectopically expressing LAP2α deletion variants as indicated on top of each lane (C) and from WT cells transfected with small interfering RNA (siRNAs) specific to BAF or non-targeting control siRNAs (#1 and #2) (D), using the E1 anti-lamin A/C antibody. Untransfected cells were included as an additional control in (D). The immunoprecipitated samples were subsequently subjected to western blot analysis using the indicated antibodies. Asterisk denotes unspecific band.

Having shown that both the N- and C-termini of LAP2α contribute to its association with chromatin, we next tested the individual contributions of the functional subdomains within these regions. We generated several LAP2α mutants: a LEM-domain mutant with mutations in the N-terminal LEM domain that are known to impair BAF binding^35^ (Figure 4A, LEM∗), a mutant that lacks the LEM-like domain (Figure 4A, ΔLEM-like), and a mutant lacking the LEM-like domain and containing the mutations in the LEM domain (Figure 4A, ΔLEM-like-LEM∗). These mutants were expressed in LAP2α KO cells. As controls, we expressed the C terminus of LAP2α (LAP2α ΔN) as well as a shorter version of the C-terminal region, each containing the lamin A/C interaction domain (Figure 4A, LAP2α C-term.).

Interestingly, normalized ChIP analyses showed that deletion of the LEM-like domain had a similarly negative effect on the association with chromatin, compared to the wild-type control, as did the deletion of the entire N terminus (Figure 4B, ΔLEM-like and ΔN mutants). In contrast, mutations in the LEM domain did not alter the association with chromatin (Figure 4B, LEM∗). Moreover, introducing the LEM mutation into the LAP2α ΔLEM-like mutant (ΔLEM-like-LEM∗) did not further reduce chromatin interaction (Figure 4B). Therefore, it is the LEM-like domain of LAP2α, rather than the LEM domain, that predominantly mediates direct association with chromatin in the N terminus.

To explain why the LEM domain of LAP2α, known to bind the DNA-crosslinking protein BAF, did not contribute to the association with chromatin of LAP2α *in vivo*, we reasoned that BAF may also interact with lamin A/C in a cellular context. This hypothesis is supported by previous reports showing strong interactions between lamin A/C and BAF in a complex with DNA *in vitro*.^36^ To test this possibility, we first assessed the binding of the LAP2α LEM and LEM-like domain mutants to lamin A/C by co-immunoprecipitation assays. Among all mutants tested, lamin A/C co-precipitated only with wild-type full-length LAP2α and the LAP2α mutant lacking the N-terminal LEM-like domain (ΔLEM-like) (Figure 4C). Intriguingly, introducing the LEM domain mutations into the ΔLEM-like mutant (ΔLEM-like-LEM∗), which impair interaction with BAF, abolished its binding to lamin A/C (Figure 4C). This indicates that the LEM domain of LAP2α might contribute to lamin A/C binding indirectly via BAF. To further test this hypothesis, we depleted BAF in wild-type cells using RNAi-mediated knockdown and analyzed the interactions of LAP2α and lamin A/C in BAF-depleted versus control cells by co-immunoprecipitation assays using a lamin A/C antibody. Strikingly, in the absence of BAF, lamin A/C failed to co-precipitate with LAP2α, suggesting that the interaction between the LEM domain and BAF is indeed crucial for the stable binding of LAP2α to lamin A/C (Figure 4D).

Overall, these results demonstrate that, in addition to the putative direct lamin A/C-binding domain in the C terminus of LAP2α,^15^ its N-terminal LEM domain contributes to a stable interaction of LAP2α and lamin A/C *in vivo*, indirectly via BAF. The interaction of a lamin A/C-BAF complex with DNA, as previously demonstrated by structural analyses,^36^ may mediate the association of lamin A/C with chromatin *in vivo*. It remains unclear whether a chromatin-associated lamin A/C-BAF complex can additionally bind LAP2α *in vivo*.

### The C terminus of LAP2α enhances chromatin association by mediating LAP2α dimerization

**Figure 5:**
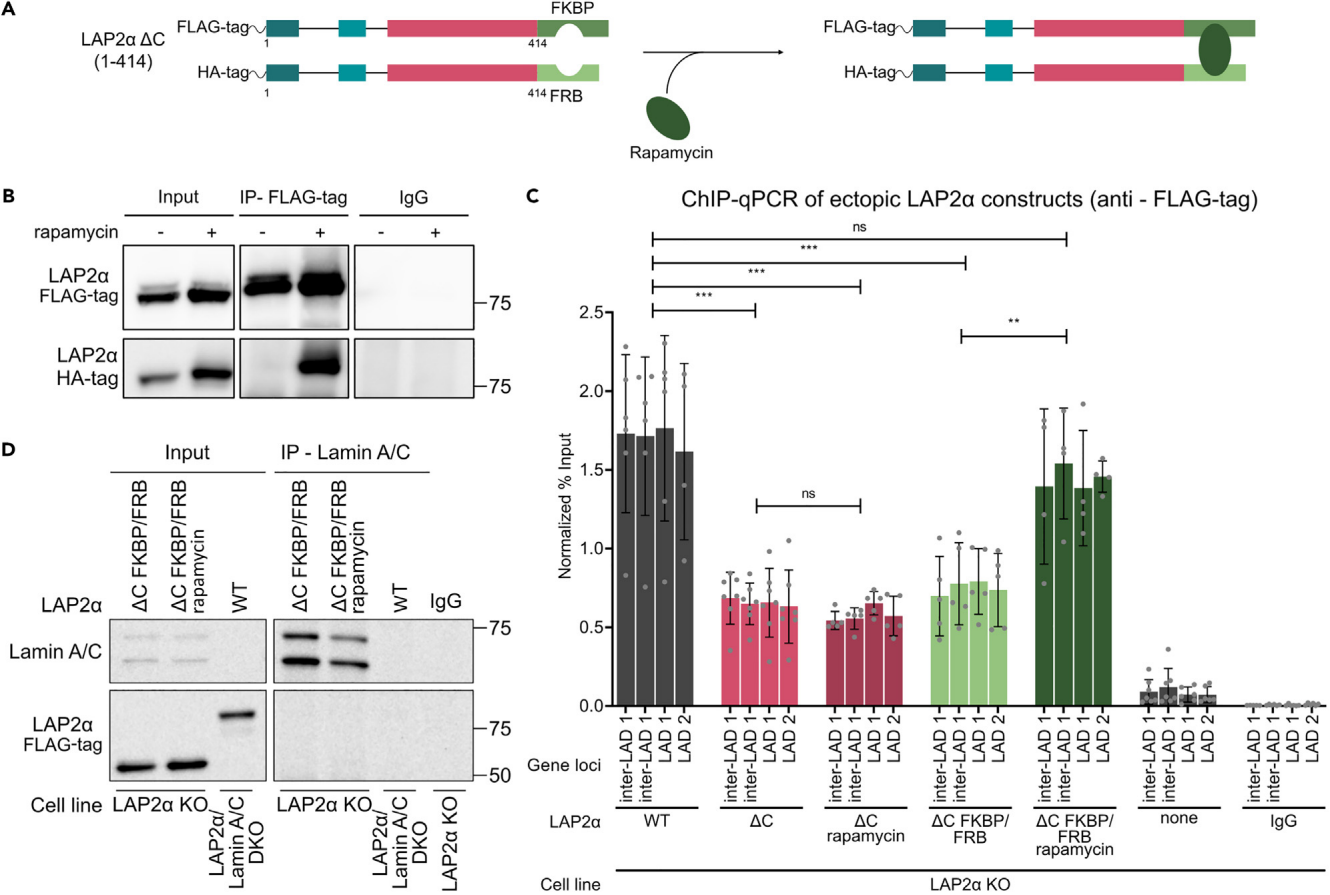
Dimerization of LAP2α is required for efficient chromatin association (A) Schematic representation of LAP2α ΔC mutants fused to FKBP and FRB tags, respectively, to employ the rapamycin-induced dimerization system. (B) FLAG-tagged LAP2α ΔC-FKBP and HA-tagged LAP2α ΔC-FRB were expressed together in LAP2α KO cells, and cells were treated for 24 h with 1 μM rapamycin or left untreated (control). FLAG-LAP2α ΔC-FKBP was then immunoprecipitated from control and rapamycin-treated cells using FLAG tag antibody. Immunoprecipitates were analyzed by western blot using the indicated antibodies. (C) Probing chromatin interaction of ectopically expressed LAP2α and its deletion mutants in LAP2α KO cells by ChIP-qPCR. ChIP was performed as described in Figure 1, using anti-FLAG antibody. Graphs display averages ±standard deviation from biological replicates. Repeated-measures one-way ANOVA test, ∗∗∗p_WT vs ΔC =_ 0.0002, ∗∗∗p_WT vs ΔC rapamycin =_ 0.0003, ∗∗∗p_WT vs ΔC FKBP/FRB_ = 0.0005, ∗∗∗p_WT vs ΔC FKBP/FRB_ = 0.0005, ns p_WT vs ΔC FKBP/FRB rapamycin_ = 0.1062, ns p_ΔC vs ΔC rapamycin_ > 0.9999, ∗∗p _ΔC FKBP/FRB vs ΔC FKBP/FRB rapamycin_ = 0.0019, p _WT_ = 6, p _ΔC_ = 7, p _ΔC rapamycin_ = 5, n _ΔC FKBP/FRB_ = 5, n _ΔC FKBP/FRB rapamycin_ = 4, p _none_ = 7, p _IgG_ = 6; F (2.087, 6.262) = 830.0. (D) Lamin A/C was immunoprecipitated from control and rapamycin-treated LAP2α KO cell lines ectopically expressing FLAG-LAP2αΔC-FKBP and HA-LAP2αΔC-FRB, using the E1 anti-lamin A/C antibody. The immunoprecipitated samples were subsequently subjected to western blot analysis using the indicated antibodies.

Having analyzed the contributions of the domains in the N-terminal region of LAP2α to chromatin association and lamin A/C binding, we next tested how the C terminus of LAP2α contributes to these interactions. Structural studies on LAP2α have shown that the protein forms a dimer, mediated by its C terminus.^34^ In order to test the significance of LAP2α dimerization for its chromatin association, we used the rapamycin-induced dimerization system. The LAP2α C terminus was replaced with protein tags containing different rapamycin-binding domains, FK506-binding protein (FKBP), or the FKBP-rapamycin-binding (FRB) domain of the mammalian target of rapamycin (mTOR) (Figure 5A). Both proteins were simultaneously expressed in LAP2α KO cells. While immunoprecipitation of FLAG-tagged LAP2α-FKBP by anti-FLAG antibody did not co-precipitate hemagglutinin (HA)-tagged LAP2α-FRB under control conditions, the addition of rapamycin mediated heterodimer formation and enabled co-precipitation of these LAP2α fusion proteins (Figure 5B). We then tested chromatin interaction of LAP2α fusion proteins in the absence and presence of rapamycin. Normalized ChIP of the fusion proteins indicated reduced association to chromatin compared to wild-type LAP2α in the absence of rapamycin (Figure 5C, ΔC FKBP/FRB), similar to mutants lacking the C terminus (LAP2α ΔC; see also Figure 3D). Strikingly, rapamycin-mediated dimerization increased chromatin association of the FKBP-fusion protein to levels of wild-type LAP2α (Figure 5C). Importantly, rapamycin addition had no effect on chromatin association of the untagged LAP2α ΔC mutant, confirming that rapamycin does not unspecifically alter chromatin binding of LAP2α. These data suggest that dimerization of LAP2α at its C terminus is essential for its chromatin interaction via the N-terminal LEM-like domain.

Additionally, in co-immunoprecipitation assays, lamin A/C failed to interact with both the monomeric and dimerized forms of the LAP2α fusion constructs (Figure 5D). These data show that rapamycin-induced dimerization of the N-terminal region of LAP2α is not sufficient to allow BAF-mediated association with lamin A/C. This supports the essential, putatively direct role of the C terminus of LAP2α in lamin A/C interaction.^15^

**Figure 6:**
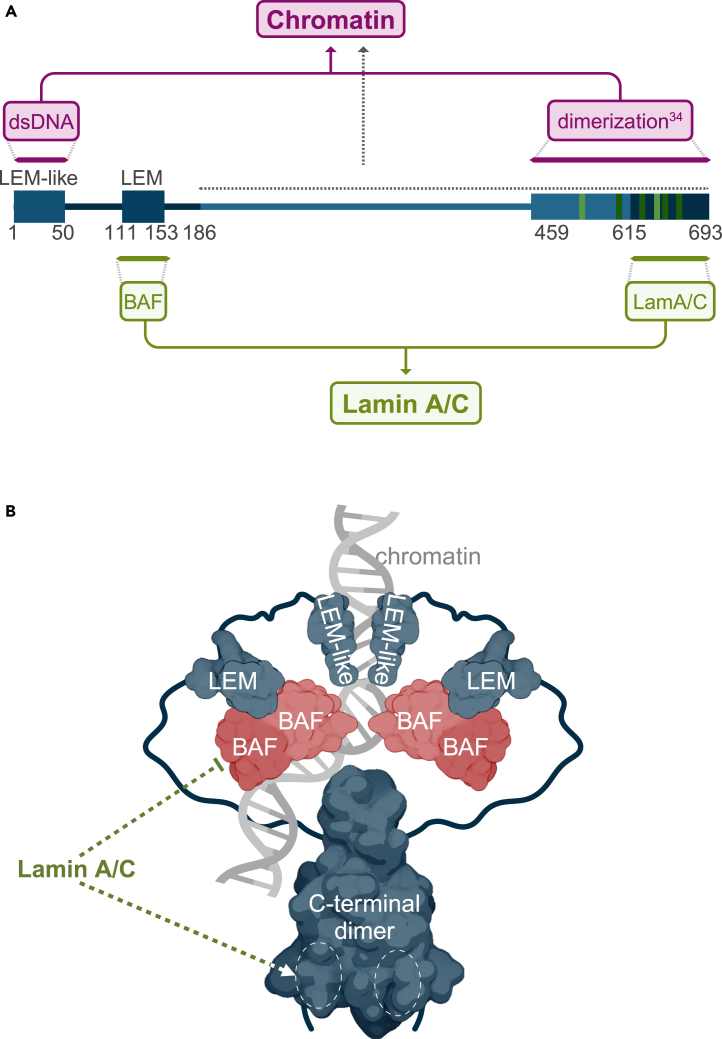
Working model (A) Schematic overview of different LAP2α domains and their functional interactions, integrating findings from this study and from published data. Bars represent the structured domains of LAP2α, while lines denote predicted unstructured regions. The LEM-like domain and the LAP2α-specific C terminus (amino acids 187–639) may contribute directly to the association of LAP2α with chromatin. Amino acids 615–693 are required for dimerization of LAPα, which is necessary for efficient interaction of the LEM-like domain with chromatin. The interaction of LAP2α with lamin A/C depends on the LEM domain, mediated by BAF, as well as on previously identified lamin A/C-binding domains in the C terminus of LAP2α.^15^^,^^34^ Amino acids 615 to 693 were found sufficient to bind lamin C *in vitro*.^15^ Mutation of residues 652–669 interfered with binding to lamin A/C^34^ (relevant residues are marked in green; mutations to Ala compromising lamin A interactions are highlighted in light green, and those enhancing interactions in darker green).^34^ (B) Conceptual model depicting the putative structure of a LAP2α dimer bound to chromatin. Two LAP2α polypeptides form dimers via their C-terminal residues. The unstructured regions upstream of the dimerization domain embrace chromatin, allowing the N-terminal LEM-like domains, and possibly the LEM domains, to interact with DNA directly and via BAF, respectively. It is unclear whether BAF, when bound to LAP2α as shown here, can still interact with lamin A/C *in vivo*. We therefore propose that the chromatin-embracing configuration of LAP2α dimers renders BAF inaccessible to lamin A/C (bar-headed dashed line), and thereby limits lamin A/C binding to weaker sites within C-terminal residues 652–669 of LAP2α dimers (indicated by arrow-headed dashed line and white dashed circles, from^34^). (B) Created with BioRender.com.

Altogether, our results support a model where a complex interplay of interactions among LAP2α, BAF, lamin A/C, and chromatin regulates the chromatin-binding properties of these proteins. LAP2α requires the N-terminal LEM-like domain, but not the LEM domain, and its dimerization, mediated by the C-terminal residues 459–693, for efficient chromatin association (Figure 6A, upper part). Moreover, additional regions within the LAP2α-specific C terminus (residues 187–693) may also contribute to chromatin binding by different mechanisms (Figure 6A, gray dashed line). This is based on our finding that LAP2α ΔN can associate with chromatin, albeit at reduced levels, in the presence of lamin A/C and even as effectively as full-length LAP2α in the absence of lamin A/C (see Figure 3D). For stable binding to lamin A/C *in vivo*, LAP2α requires cooperation of its N-terminal LEM domain, which may bind lamin A/C indirectly via BAF, and regions in the very C terminus, which were shown to interact with lamin A/C directly *in vitro* (Figure 6A, lower part).

Interestingly, chromatin association of full-length LAP2α is not affected by the presence or absence of lamin A, while the presence of LAP2α weakens lamin A/C chromatin binding. These findings may be explained by the existence of different protein complexes containing or lacking LAP2α, lamin A/C, and BAF. A complex of LAP2α and BAF may interact with chromatin independently of lamin A/C. We envisage a LAP2α dimer that embraces chromatin via its unstructured regions and binds DNA through its LEM-like domain and possibly via BAF bound to the LEM domain, which may in turn restrict the interaction of lamin A with BAF (Figure 6B). Chromatin-bound LAP2α complexes may limit the access of lamin A/C-BAF complexes to chromatin. Additionally, a soluble non-chromatin-bound LAP2α-BAF complex in the nucleoplasm may recruit a fraction of chromatin-bound lamin A/C to these complexes, thereby reducing lamin A/C chromatin interaction (see graphical abstract). Overall, these findings underscore the intricate regulatory mechanisms governing the dynamic chromatin interactions of LAP2α and lamin A/C.

## Discussion

Our findings reveal a complex interplay between LAP2α and A-type lamins in the regulation of their interactions with both hetero- and euchromatin. LAP2α and lamin A/C complexes bind to chromatin independently. Furthermore, chromatin-bound LAP2α likely competes with lamin A/C for binding sites on chromatin, as indicated by the increased association of A-type lamins with chromatin in the absence of LAP2α. Additionally, our study reveals a complex collaboration among different LAP2α domains, facilitating efficient association with chromatin and stable interaction with lamin A/C. Our findings also suggest that LAP2α can recruit lamin A/C into a soluble complex that is not associated with chromatin.

The functions of A-type lamins within the peripheral nuclear lamina are well characterized,^5^ yet the regulatory mechanisms and specific roles of the nucleoplasmic A-type lamin pool remain elusive.^9^ LAP2α, a lamin A/C interacting protein, is a key modulator of lamin A/C properties and functions in the nucleoplasm.^10^^,^^15^^,^^21^^,^^22^^,^^23^ Despite considerable progress in the past years, the mechanisms by which LAP2α influences chromatin association of lamin A/C are not fully understood. Here we report that lamin A/C showed a stronger and/or more stable association with hetero- and euchromatin in LAP2α-depleted versus wild-type cells, implying an inhibitory role of LAP2α in chromatin association of lamin A/C. In contrast, the absence of A-type lamins did not affect chromatin association of full-length LAP2α. One hypothesis to explain these findings is that the loss of LAP2α promotes the formation of stable, possibly higher-order assembly structures of lamin A in the nucleoplasm.^10^ These changes in lamin A/C properties may also cause changes in chromatin association of lamin A/C. However, an assembly-deficient phosphomimetic S/D lamin A mutant^26^ associated with chromatin as efficiently as assembly-competent lamin A variants, indicating that the assembly state of lamins does not affect their chromatin association. This also suggests that the changes in the association of lamin A/C with chromatin upon LAP2α loss are not driven by modulation of lamin assembly states. Furthermore, our studies revealed that LAP2α-mediated effects on the association of lamin A/C with chromatin do not require the formation of stable complexes of these proteins *in vivo*. Accordingly, expression of lamin binding-deficient LAP2α fragments in LAP2α-depleted cells decreased lamin A/C chromatin association to wild-type levels, similar to ectopic full-length LAP2α.

To gain further insight into how LAP2α may affect the association of lamin A/C with chromatin, we analyzed in detail the interplay of different LAP2α domains, which were previously described to be involved in chromatin and/or lamin interactions *in vitro*. Two structured regions in the N-terminal 186 residues of LAP2α, a helix-loop-helix-type LEM domain and an LEM-like motif,^18^^,^^19^ mediate DNA interactions indirectly through BAF and directly by binding to DNA, respectively.^18^^,^^35^ A C-terminal region spanning residues 459–693 is proposed to form dimers, possessing an extensive four-stranded, antiparallel coiled coil, and was shown to interact with A-type lamins *in vitro*.^15^^,^^34^ While the roles of these individual domains in chromatin association and lamin interactions are well documented *in vitro*, it remains elusive whether they contribute to chromatin binding of LAP2α *in vivo* and how these interactions may mutually influence each other.

Here, we show that LAP2α associates with hetero- and euchromatin independently of lamin A/C. Efficient chromatin interaction of LAP2α requires both the N-terminal LEM-like domain and its C-terminal region. As the C terminus of LAP2α was proposed by structural studies to form dimers,^34^ we tested whether dimerization enhances chromatin association of LAP2α per se, or whether the C terminus contributes to chromatin binding of LAP2α by other means. Using a rapamycin-inducible dimerization system, where the C-terminal dimerization domain of LAP2α was replaced by rapamycin-dependent interaction domains, we confirmed that dimerization of LAP2α via its C-terminal domain is necessary for its efficient association with chromatin. We hypothesize that LAP2α dimerization at its C terminus may mediate strong chromatin association of the two N-terminal LEM-like domains in the LAP2α dimer. Additionally, the entire LAP2α-specific C terminus (residues 187–693) has some residual chromatin association activity, as it associates weakly with chromatin in lamin A/C-expressing cells and displays chromatin binding at the level of wild-type LAP2α in lamin A/C-deficient cells. This led us to conclude that the C-terminal domain of LAP2α and lamin A/C compete for binding to chromatin. In contrast, chromatin association of full-length LAP2α is not affected by lamin A/C, likely due to the strong binding of the LEM-like domains to chromatin.

In addition to its role in dimerization, the C terminus of LAP2α was shown to be sufficient for interaction with A-type lamins *in vitro*.^15^^,^^34^ It is unclear, however, whether dimerization and lamin A/C binding are mutually exclusive or occur simultaneously within one complex. Surprisingly, our studies revealed that the interaction of the C terminus of LAP2α with lamin A/C is not detectable *in vivo* in co-immunoprecipitation analyses. Instead, stable interaction of these proteins also requires the presence of the N-terminal LEM domain of LAP2α, which likely contributes to lamin A/C binding indirectly via BAF. If and how the BAF-mediated interaction of the LEM domain with lamin A influences its BAF-mediated interaction with chromatin remains elusive. A recent study solved the X-ray structure of a ternary complex consisting of a BAF dimer and protein fragments representing the LEM-domain and the immunoglobulin (Ig) domain of lamin A.^36^ Additionally, they aligned the three-dimensional model of this ternary complex with the previously resolved X-ray structure of a BAF dimer in complex with DNA.^37^ This superimposition led the authors to propose that BAF is capable of simultaneously interacting with the LEM domain, the Ig domain of lamin A, and DNA; however, this has not yet been confirmed experimentally. Based on these *in vitro* data, it is possible that a similar complex of LAP2α, BAF, lamin A/C, and chromatin exits *in vivo*, in which LAP2α employs its LEM-like domain to interact with DNA, while its LEM domain and C terminus are bound to BAF and lamin A/C. However, our observation that lamin A/C depletion does not affect LAP2α chromatin association makes this scenario less likely.

Instead, considering all our findings, we favor a model in which various protein complexes composed of different combinations of LAP2α, lamin A/C, BAF, and possibly other proteins may either associate with chromatin or exist as a dynamic soluble pool in the nucleoplasm (see graphical abstract). Based on the observation that both LAP2α and lamin A/C can bind to chromatin independently of each other, we hypothesize that LAP2α-BAF and lamin A/C-BAF complexes may associate with chromatin separately. The observation that the presence of LAP2α impairs chromatin association of lamin A/C *in vivo* is consistent with at least two, not mutually exclusive, scenarios. Firstly, association of LAP2α-BAF complexes with chromatin may hinder efficient binding of lamin A-BAF complexes to chromatin. Secondly, LAP2α in the nucleoplasm may recruit lamin A/C from chromatin into dynamic, soluble nucleoplasmic protein complexes (see graphical abstract). The latter scenario is consistent with our observation that LAP2α, lamin A/C, and BAF coprecipitate from a soluble, chromatin-depleted cell lysate, and with our previous findings that LAP2α maintains a soluble, relatively mobile pool of lamin A/C in the nuclear interior.^10^

An additional mechanism to consider is that LAP2α and/or lamin A/C complexes may form liquid-liquid phase-separated compartments on chromatin, based on the presence of intrinsically disordered low-complexity regions in large parts of the LAP2α polypeptide.^38^ These compartments may attract or exclude lamin A/C, BAF, and other regulatory proteins under different conditions. This could in turn regulate transcription and other DNA-linked processes. While this model represents an attractive mechanism for chromatin regulation by LAP2α and lamin A/C, it is purely speculative at this stage requiring extensive experimental testing in subsequent studies.

Regarding the physiological significance of a LAP2α-mediated regulation of the association of lamin A/C with chromatin, previous studies indicated crucial roles of LAP2α in early differentiation stages of progenitor and stem cells across different tissues.^23^^,^^39^ LAP2α is required to facilitate the transition of tissue progenitor cells from a proliferation state to a quiescent or differentiation state.^23^^,^^39^^,^^40^^,^^41^ The inhibition of the association of lamin A/C with euchromatin by LAP2α may be particularly important for limiting lamin A/C binding to actively transcribed genomic regions during cell state transitions. Restriction of lamin A/C binding to these regulatory genomic regions may be crucial, as lamin accumulation could potentially block access for transcription factors and gene regulatory proteins, thereby influencing tissue-specific gene regulation.

In summary, our study tackles the extremely complex and interdependent regulation of LAP2α and lamin A/C in the nucleoplasm and on chromatin at a mechanistic level. We demonstrate the vital roles of both the N- and C-terminal domains of LAP2α in its association with chromatin and its interaction with lamin A/C, as well as their contributions to the regulation of lamin A/C chromatin binding. Our insights into these interactions contribute to a deeper understanding of nucleoplasmic lamins and their role in nuclear and chromatin organization and might have implications for chromatin and gene expression abnormalities frequently observed in several lamin-linked diseases.

### Limitations of the study

Our analysis of ectopically expressed lamin assembly-deficient mutants focused exclusively on lamin A. We therefore cannot exclude that lamin C may behave differently. This is particularly relevant, as recent studies reported different functions of lamins A and C. Lamin C, rather than lamin A, was found to be essential for the 3D organization of LADs during mitotic exit and for overall chromosome organization.^42^ Furthermore, phosphorylated lamin C bound strongly to enhancers in euchromatin,^33^ and lamin C rather than lamin A seemed to accumulate at sites of nuclear rupture rapidly to initiate repair.^43^ Additionally, chromatin association analyses of LAP2α and lamin A/C in this study were limited to four genomic loci, two within heterochromatic and two within euchromatic genomic regions, and did not involve genome-wide experiments, which may limit the generalizability of our findings.

## Resource availability

### Lead contact

Further information and requests for resources and reagents should be directed to the lead contact, Roland Foisner (roland.foisner@meduniwien.ac.at).

### Materials availability

This study did not generate new unique reagents.

### Data and code availability

• Data: the raw qPCR results, original microscopy, and western blot images (Figures 1, 2, 3, 4, 5, S2, and S4) have been deposited at Mendeley and are publicly available as of the date of publication. The DOI is listed in the key resources table.
• Code: no large datasets were generated or analyzed during the current study.
• Any additional information required to reanalyze the data reported in this paper is available from the lead contact upon request.

## Acknowledgments

Microscopic analyses and FACS sorting were performed in the BioOptics facility at the Max Perutz Labs. This research was funded in whole or in part by the 10.13039/501100002428Austrian Science Fund (FWF) (P32512-B and P36503-B) to R.F. and a doctorate program funded by the 10.13039/501100002428Austrian Science Fund (FWF) (W1261-B28). For open access purposes, the author has applied a CC BY public copyright license to any author-accepted manuscript version arising from this submission. K.G. and D.F. are recipients of a DOC Fellowship of the Austrian Academy of Sciences at the Max Perutz Labs, Medical University Vienna (ÖAW DOC 25725 and ÖAW DOC 25912, respectively). This work was supported by the Marie Jahoda fellowship of the University of Vienna to N.N.

The graphical abstract and Figure 6B were created with BioRender.com.

## Author contributions

Conceptualization, D.F., R.F., and N.N.; performance of the experiments, D.F. and A.S.; methodology, D.F., N.N., and K.G.; data interpretation and writing the manuscript, D.F., R.F., and N.N.; funding acquisition, R.F. and N.N.; supervision, R.F. and N.N.

## Declaration of interests

The authors declare no competing interests.

## STAR★Methods

### Key resources table

**Table:** 

REAGENT or RESOURCE	SOURCE	IDENTIFIER
**Antibodies**		

Anti-lamin A/C E1 (mouse, monoclonal)	Santa Cruz Biotechnology	Cat# sc-376248; RRID: AB_10991536
Anti-lamin A/C 3A6 (mouse, monoclonal)	Max Perutz Labs Monoclonal Antibody Facility, see also^21^	N/A
Anti-FLAG M2 (mouse, monoclonal)	Sigma Aldrich	Cat# F3165; RRID: AB_259529
Anti-HA rabbit (rabbit, polyclonal)	Sigma-Aldrich	Cat# H6908; RRID: AB_260070
Anti-BANF1 M2 (mouse, monoclonal)	Novus Biologicals	Cat# H00008815-M07; RRID: AB_537085
Anti-lamin B1 (rabbit, polyclonal)	Proteintech	Cat# 12987–1-AP; RRID: AB_2136290
Anti-LAP2α 245.2 (rabbit, polyclonal)	Max Perutz Labs Monoclonal Antibody Facility, see also^44^	N/A
Anti-LAP2α 1H11 (mouse, monoclonal)	Max Perutz Labs Monoclonal Antibody facility, see also^21^	N/A
Anti-γ-tubulin GTU88 (mouse, monoclonal)	Sigma Aldrich	Cat# T6557; RRID: AB_477584
IgG (mouse, polyclonal)	Sigma Aldrich	Cat# 12–371; RRID: AB_145840

**Chemicals, peptides, and recombinant proteins**		

DAPI-containing mounting medium Vectashield	Vector Laboratories	Cat# H-1200-10
Complete Protease Inhibitor Cocktail	Sigma-Aldrich	Cat# 11697498001
benzonase	Millipore	Cat# 70746
Pierce Protein A/G Magnetic Beads	Thermo Fisher Scientific	Cat# 88803
Pierce 16% Formaldehyde (w/v), Methanol-free	Thermo Fisher Scientific	Cat# 28908
RNase A, DNase and protease-free	Thermo Fisher Scientific	Cat# EN0531
Proteinase K, recombinant, PCR grade	Thermo Fisher Scientific	Cat# EO0491

**Critical commercial assays**		

NE-PER Nuclear and Cytoplasmic Extraction Reagents	Thermo Fisher Scientific	Cat# 78833
SuperSignal West Pico PLUS Chemiluminescent Substrate	Pierce/Thermo Fisher Scientific	Cat# 34577
Qubit 1X dsDNA Broad Range (BR) Assay Kit	Thermo Fisher Scientific	Cat# Q33265
ChIP DNA Clean & Concentrator kit	Zymo Research	Cat# D5205
KAPA SYBR FAST	Roche	Cat# KK4618
Q5 Site-Directed Mutagenesis Kit	New England Biolabs	Cat# E0554S

**Deposited data**		

All original raw data generated for this paper	This paper; Mendeley Data	https://doi.org/10.17632/m42wvvf7ns.1

**Experimental models: Cell lines**		

LAP2α wildtype (WT) mouse dermal fibroblasts	This lab	N/A
LAP2α knockout (KO) mouse dermal fibroblasts	This lab	N/A
LAP2α/lamin A/C double knockout (DKO) mouse dermal fibroblasts	This lab	N/A

**Oligonucleotides**		

ON-TARGETplus Non-targeting Control siRNAs, Pool	Dharmacon	Cat# D-001810-10-05
ON-TARGETplus Non-targeting Control siRNAs, Control#4	Dharmacon	Cat# D-001810-04-05
ON-TARGETplus Mouse Banf1 siRNA smartpool	Dharmacon	Cat# L-062803-01-0005
See Table S3 for primers references used in this study	This paper	N/A

**Recombinant DNA**		

pLVX mCherry plasmid	Takara Bio	Cat# 631987
pLVX puro plasmid	Takara Bio	Cat# 631847
CFP-PCRD-FRB	Addgene	Cat# 87449
FKBP-DCRD-YFP	Addgene	Cat# 87450

**Software and algorithms**		

GraphPad Prism version 10.0.0 for Windows	GraphPad Software, Boston, Massachusetts USA	www.graphpad.com
Image Lab Software for PC Version 6.1	Bio-Rad Laboratories, Inc.	N/A

**Other**		

Zeiss LSM 980 confocal microscope, equipped with a Plan-Apochromat 63×/1.4 Oil DIC WD 0.19 mm objective and standard photomultiplier tubes (PMTs) for sequential detection	Zeiss	N/A
Bandelin Sonopuls HD200 sonicator	Bandelin	N/A
Bioruptor Pico sonication device	Diagenode	N/A
tubes and sonication beads	Diagenode	Cat# C01020031
Qubit 4	Thermo Fisher Scientific	N/A
nitrocellulose membranes with 0.2 μm pore size	Cytiva	Cat# GE10600001

### Experimental model and study participant details

#### Cell lines

LAP2α wildtype (WT), LAP2α knockout (KO), and LAP2α/lamin A/C double knockout (DKO) mouse dermal fibroblasts, generated using CRISPR/Cas9 as described in Naetar et al,^10^ were maintained in Dulbecco’s modified Eagle’s medium (DMEM) supplemented with 10% fetal calf serum (FCS), 2 mM glutamine, 100 U/mL penicillin, 100 μg/mL streptomycin (P/S) (all from Sigma-Aldrich) and non-essential amino acids (PAN-Biotech) in a humidified incubator at 37°C with 5% CO2.

### Method details

#### Immunofluorescence

Cells were seeded on uncoated glass coverslips (1.5H, Marienfeld-Superior), followed by fixation with 4% paraformaldehyde for 10 min at room temperature. To quench the fixation and permeabilize the cells, samples were incubated in PBS with 0.1% Triton X-100 and 50 mM NH_4_Cl for 5 min at room temperature. Subsequently, cells were incubated with primary (anti-lamin A/C 3A6 1:50, anti-FLAG 1:100, anti-LAP2α 245.2^44^ 1:800) and secondary antibodies for 1 h at room temperature. Finally, cells were mounted and DNA was stained with the DAPI-containing mounting medium Vectashield (Vector Laboratories). Imaging was performed using the Zeiss LSM 980 confocal microscope, equipped with a Plan-Apochromat 63×/1.4 Oil DIC WD 0.19 mm objective and standard photomultiplier tubes (PMTs) for sequential detection. Image acquisition was done using Zeiss ZEN 3.3 software, and image processing was carried out with FIJI ImageJ software.

#### Biochemical extraction of proteins

Cells cultured on dishes were first washed twice with ice-cold PBS buffer, and then harvested in PBS containing 1× Complete Protease Inhibitor Cocktail (Sigma-Aldrich) using a cell scraper. The cells were subsequently centrifuged at 4°C for 4 min at 1,400 rpm using a Megafuge 1.0R (Haereus). After discarding the supernatant, the cell pellets were resuspended in extraction buffer (20 mM Tris-HCl pH 7.5, 150 mM NaCl, 2 mM EGTA, 2 mM MgCl_2_, 0.5% NP-40, 1 mM DTT, and 1× Complete Protease Inhibitor Cocktail from Sigma-Aldrich) and lysed for 10 min on ice. Afterward, the lysates were centrifuged at 4°C for 10 min at 4,000 rpm using a Megafuge 1.0R (Haereus). The resulting pellets were resuspended in extraction buffer using volumes equal to the soluble supernatant fraction and sonicated with a Bandelin Sonopuls HD200 sonicator (Bandelin; settings: MS73/D at 50% intensity) for 3 s to solubilize insoluble material. The total (before centrifugation), supernatant, and solubilized pellet fractions were analyzed by immunoblotting. Nuclear and cytoplasmic protein extraction was conducted using NE-PER reagents (Thermo Fisher Scientific) according to the manufacturer’s protocol. Subsequently, the samples were analyzed by immunoblotting.

#### Immunoprecipitation and immunoblotting

For co-immunoprecipitation, cells were scraped off the plate in immunoprecipitation (IP) buffer (20 mM Tris-HCl, pH 7.5, 150 mM NaCl, 2 mM EGTA, 2 mM MgCl_2_, 0.5% NP-40, 1 mM DTT, 1 U/mL Benzonase from Millipore, 1× Complete Protease Inhibitor cocktail from Sigma-Aldrich), followed by a 10-min lysis on ice. Protein extracts were then centrifuged at 4°C for 10 min at 4,000 rpm in a Megafuge 1.0R (Haereus). The supernatant, containing the soluble fraction of proteins, was pre-cleared by incubation with protein A/G dynabeads (Pierce/Thermo Fisher Scientific) for 20 min at 4°C. Pre-cleared extracts were then incubated with antibodies (5 μg/IP) overnight on a rotator. Protein-antibody complexes were pulled down using BSA-blocked protein A/G dynabeads for 4 h at 4°C, followed by three 5-min washing steps at 4°C in IP buffer without benzonase on a rotator. Finally, immunoprecipitated protein complexes were eluted from the beads in SDS PAGE sample buffer for subsequent immunoblot analysis.

For immunoblotting, protein extracts were separated on SDS polyacrylamide gels, and proteins were transferred onto nitrocellulose membranes with 0.2 μm pore size (Cytiva). Membranes were then blocked in 5% milk in PBST (PBS with 0.05% Tween 20) and incubated with primary antibodies (anti-lamin A/C E1 1:1000, anti-FLAG 1:1000, anti-HA 1:1000, anti-BANF1 1:500, anti-lamin B1 1:500, anti-GAPDGH 1:10,000, anti-LAP2α 1H11 1:100, anti-γ-tubulin 1:5000) overnight at 4°C, and with horseradish peroxidase-coupled secondary antibodies (anti-mouse secondary antibody 1:15,000 or anti-rabbit secondary antibody 1:30,000) for 1.5 h at room temperature. Signal detection was performed using SuperSignal West Pico Plus chemiluminescent substrate (Pierce/Thermo Fisher Scientific) and the ChemiDoc Gel Imaging system (Bio-Rad). Image analysis and quantification were carried out using the Image Lab software (Bio-Rad).

#### Chromatin immunoprecipitation with quantitative polymerase chain reaction (ChIP-qPCR)

Cells were seeded on 15 cm cell culture dishes 24 h before harvesting. For harvesting, cells were trypsinized, followed by neutralization with 4 volumes of FBS-containing medium, washing with PBS, and resuspension in PBS at a concentration of 2 × 10^6^ cells/mL. Formaldehyde fixation (62.5 μL of 16% formaldehyde per 2 × 10^6^ cells) was carried out for 10 min at room temperature while rotating at 8 rpm. Glycine was then added to quench the reaction (50 μL of 2.5M glycine/mL crosslinking reaction), and after 5 min of rotation at 8 rpm, cells were washed twice with ice-cold PBS, with a centrifugation step for 5 min at 1,500 rpm (Megafuge 1.0R, Haereus) after each wash. The pellet was then resuspended in ice-cold Wash 1 Buffer (0.25% Triton X-100, 10 mM EDTA, 0.5 M EGTA, 10 mM HEPES, 1× Complete Protease Inhibitor cocktail from Sigma-Aldrich, 0.1 mM PMSF) at a concentration of 2 × 10^6^ cells/mL buffer and incubated for 10 min on ice. After a 5-min centrifugation at 1,500 rpm (Megafuge 1.0R, Haereus) and 4°C, the pellet was resuspended in ice-cold Wash 2 Buffer (200 mM NaCl, 1 mM EDTA, 0.5 mM EGTA, 10 mM HEPES, 1× Complete Protease Inhibitor cocktail from Sigma-Aldrich, 0.1 mM PMSF) at a concentration of 2 × 10^6^ cells/mL buffer and centrifuged for 5 min at 1,500 rpm (Megafuge 1.0R, Haereus) and 4°C. Next, the pellet was resuspended in Lysis Buffer (1% SDS, 10 mM EDTA, 50 mM Tris-HCl, pH 8.1, 1× Complete Protease Inhibitor cocktail from Sigma-Aldrich, 0.1 mM PMSF) at a concentration of 1 × 10^7^ cells/mL.

Chromatin sonication was performed using a Bioruptor Pico sonication device (Diagenode) for 6 cycles of 30 s ON/30 s OFF in 15 mL Bioruptor sonication tubes containing 500 mg of sonication beads (both from Diagenode). After sonication, chromatin was centrifuged for 15 min at 15,000 rpm and 4 °C (Centrifuge 5417 R, Eppendorf), and the supernatant was diluted in Dilution Buffer (167.4 mM NaCl, 16.72 mM Tris-HCl, pH 8.1, 1.2 mM EDTA, 1.1% Triton X-100, 0.001% SDS, 1× Complete Protease Inhibitor cocktail from Sigma-Aldrich) in a 1:0.5 ratio. The DNA concentration was measured using Qubit 4 and the broad range dsDNA kit (both from Thermo Fisher Scientific) following the manufacturer’s protocol.

For chromatin immunoprecipitation, the equivalent of 6 × 10^6^ cells/ChIP were diluted in 1.6 mL Dilution Buffer and incubated with antibodies (anti-lamin A/C E1 36 μL/ChIP, anti-lamin A/C 3A6 90 μL/ChIP, anti-FLAG M2 4.5 μL/ChIP, anti-LAP2α 1H11 180 μL/ChIP, IgG 1.5 μL/ChIP) overnight at 4°C while rotating at 12 rpm. Input samples (10% of the total volume) were also incubated with the ChIP samples overnight. 30 μL of ProteinA/G dynabeads (Pierce/Thermo Fisher Scientific) were added to the ChIP samples and incubated for 4 h at 4°C while rotating at 12 rpm. After incubation, beads were washed with the following buffers in this order: RIPA Buffer (150 mM NaCl, 50 mM Tris-HCl, pH 8.0, 0.1% SDS, 0.5% Sodium Deoxycholate, 1% NP-40), High Salt Buffer (500 mM NaCl, 50 mM Tris-HCl, pH 8.0, 0.1% SDS, 1% NP-40), LiCl Buffer (250 mM LiCl, 50 mM Tris-HCl, pH 8.0, 0.5% Sodium Deoxycholate, 1% NP-40), and twice with Tris-EDTA (TE) Buffer (10 mM Tris-HCl, pH 8.0, 1 mM EDTA).

To elute the chromatin, the beads were resuspended in 200 μL Elution Buffer (100 mM NaHCO_3_, 2% SDS, 10 mM DTT) and incubated for 30 min at room temperature, while shaking at 1,200 rpm. Elution buffer was also added to the input samples and from this point on inputs were processed as the immunoprecipitated samples. The supernatant containing the chromatin eluted from the beads was transferred to a new 1.5-mL microcentrifuge tube, 10 μL of 4M NaCl/200 μL sample was added, and samples were incubated overnight at 65°C while shaking at 300 rpm for decrosslinking. Samples were then treated with 4 μL 0.5M EDTA, 8 μL 1M Tris-HCl, pH 6.5, and 8 μg of RNase A (always per 200 μL sample) for 1 h at 37°C while shaking at 300 rpm. Next, samples were incubated with Proteinase K at a final concentration of 250 μg/mL for 2 h at 55°C and shaking at 300 rpm. Finally, DNA was purified with the ChIP DNA Clean & Concentrator kit (Zymo Research) following the manufacturer’s protocol. The collected samples were analyzed by qPCR using the KAPA SYBR Green 2× PCR master mix (Kapa Biosystems) and primers designed for genomic loci previously identified as lamin A/C and LAP2α binding sites^21^ (see Table S3 and Figure S2). The analysis was conducted using an Eppendorf Realplex 2 Mastercycler following the guidelines provided by the manufacturer. To account for batch-to-batch variability, the % input was additionally normalized by calculating the average % input across all samples of the relevant replicate for each specific genomic locus separately. The initial % input value for each sample and genomic locus obtained from the ChIP experiment was then divided by the respective average, resulting in % input ratios that were subsequently employed for statistical analyses. For analyses of ChIP signals of ectopically expressed LAP2α mutants, these % input values were normalized to the relative nuclear protein levels identified by Western blot analyses of nuclear fractions by dividing the % input values by respective relative amounts (see Figure S2 and S4).

#### Vectors

The pLVX mCherry plasmid was used to express FLAG-tagged LAP2α or lamin A constructs. For the LAP2α ΔC-FRB mutant, we utilized the pLVX puro plasmid (both plasmids from Takara Bio). The pLVX mCherry plasmid was digested with EcoRI and XbaI, and the pLVX puro plasmid with EcoRI and BamHI. Subsequently, the final vectors containing the desired constructs were generated by assembling multiple fragments using the Gibson assembly master mix (New England Biolabs). Fragments were obtained by PCR using primers and templates listed in Tables S1 and S3.

To generate pLVX mCherry plasmids containing LAP2α LEM∗, lamin An S22/392D, or lamin An S22/392A, the Q5 Site-Directed Mutagenesis kit (New England Biolabs) was used according to the manufacturer’s protocol. The specific mutations were introduced using primer pairs and templates listed in Tables S2 and S3.

### Quantification and statistical analysis

All data were analyzed using GraphPad Prism version 10.0.0 for Windows (GraphPad Software, Boston, Massachusetts USA, www.graphpad.com). Statistical analyses were done using a minimum of three biological replicates. The specific number of replicates for each experiment is indicated in the respective figure legend as number of n. Normal distribution of the data was assessed using the D’Agostino-Pearson and Shapiro-Wilk normality tests. Additionally, quantile-quantile plots were generated and visually inspected to verify normal distribution. For datasets demonstrating normal distribution with paired data points, the paired two-tailed Student’s t test was employed for statistical analysis. Ratios were subjected to logarithmic transformation in order to obtain a normally distributed dataset. When comparing more than two datasets, repeated measures (RM) one-way ANOVA was utilized, followed by post-hoc pairwise multiple comparison test (Sidak). The specific statistical tests and corresponding asterisks for each graph are indicated in the respective figure legends. The following *p*-value ranges were used for statistical significance, represented by asterisks: ns (*p* > 0.05); ∗ (*p* ≤ 0.05); ∗∗ (*p* ≤ 0.01); ∗∗∗ (*p* ≤ 0.001); ∗∗∗∗ (*p* ≤ 0.0001).
