# Supplementary material for: Lamin chromatin binding is modulated by interactions of different LAP2α domains with lamins and chromatin
Source: iScience. 2024 Sep 2;27(10):110869. doi: 10.1016/j.isci.2024.110869 (PMC11417337; doi:10.1016/j.isci.2024.110869)
Supplement: Document S1. Figures S1–S4 and Tables S1–S3 [file mmc1.pdf]

iScience, Volume *27*

Supplemental information

Lamin chromatin binding is modulated by interactions of different LAP2α domains with lamins and chromatin

Daria Filipczak, Anna Souchet, Konstantina Georgiou, Roland Foisner, and Nana Naetar

Supplemental information

Supplementary Figures and Legends: Figures S1–S4

Tables S1-S3

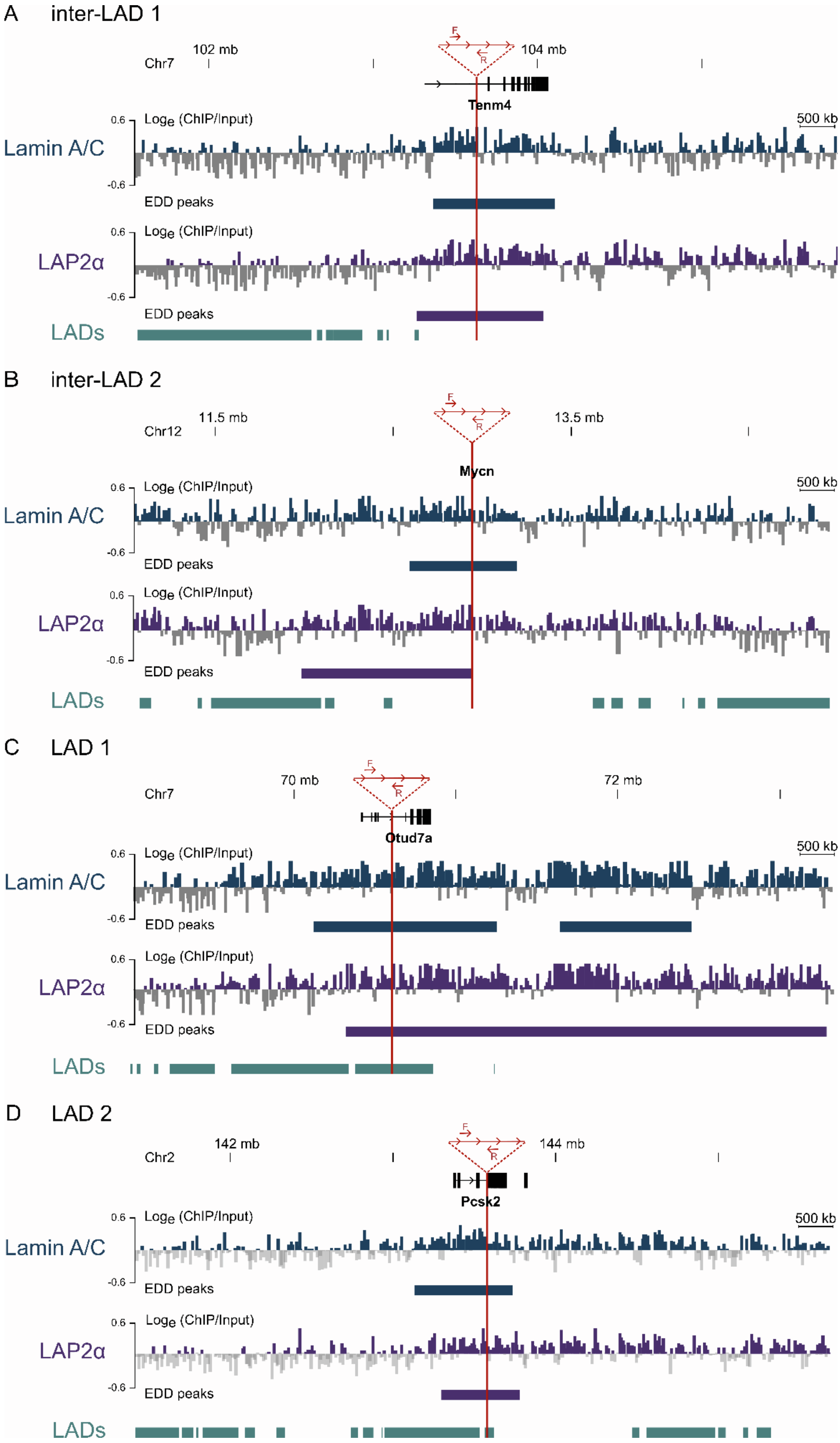
A inter-LAD 1
Chr7
102 mb
104 mb
F
R
Tenm4
Lamin A/C
0.6
-0.6
Loge (ChIP/Input)
500 kb
EDD peaks
LAP2α
LADs
B inter-LAD 2
Chr12
11.5 mb
13.5 mb
Mycn
C LAD 1
Chr7
70 mb
72 mb
Otud7a
D LAD 2
Chr2
142 mb
144 mb
Pcsk2

Figure S1. **Integrative Genomics Viewer (IGV) tracks showing genomic regions analyzed in lamin A/C and LAP2α ChIP. Related to: Figures 1–5. (A-D)** Genomic loci studied in ChIP-qPCR were defined from previously published LAP2α and lamin A/C ChIP-seq analysis [S1]. ChIP-seq analysis was performed in wildtype immortalized mouse dermal fibroblasts, using lamin A/C antibody 3A6 (lamin A/C; blue) and LAP2α antibody 1H11 (LAP2α; purple). Log2 ratio tracks in the IGV browser show regions of chromosome 7 (A,C), chromosome 12 (B) and chromosome 2 (D). Enriched Domain Detector (EDD) software was used to call peaks. Positive log2 ratio values are depicted in color, negative values in grey. The scale of log2 ratio tracks is indicated on the left. Genes overlapping with the area selected for PCR amplification (red line) are depicted on top of each panel. cLADs: constitutive lamina-associated domains.

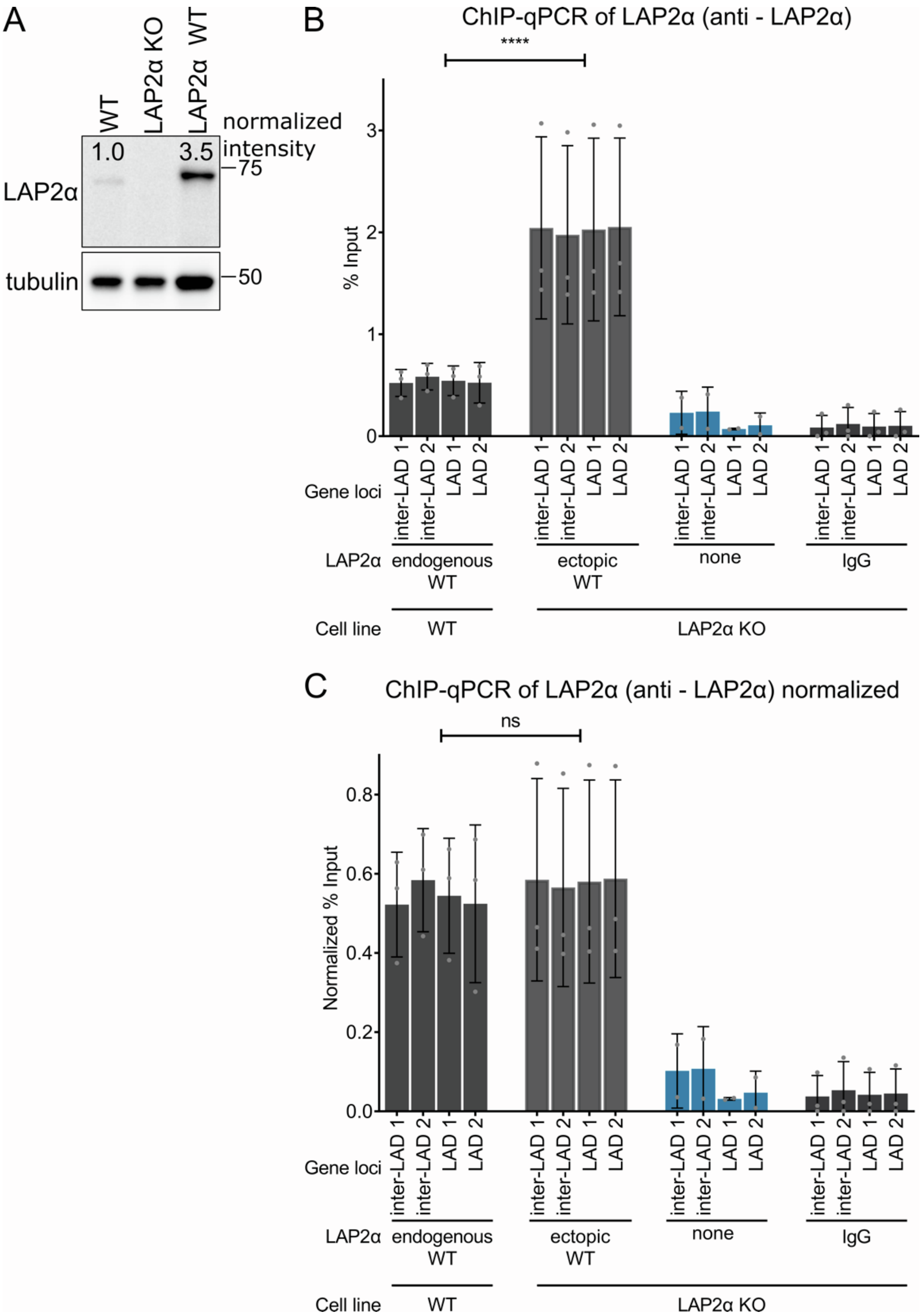

Figure S2. **Expression levels of LAP2α correlate with ChIP signal measured by qPCR. Related to Figures 3–5 and STAR Methods. (A)** Protein extracts of wildtype (WT) and LAP2α KO cells and of LAP2α KO cells ectopically expressing wildtype LAP2α (LAP2α WT) were analyzed by Western blot using the indicated antibodies. Western blot signals for LAP2α were quantified, normalized to tubulin, and relative

values (fold difference to the wildtype sample) are shown above each band. **(B, C)** Probing chromatin interaction of endogenous LAP2α and ectopically expressed LAP2α WT in LAP2α KO cells using ChIP-qPCR. Graphs depict ChIP results before (b) and after (c) normalization to LAP2α expression level as determined in (a). ChIP was performed as described in Fig. 1, using 1H11 anti-LAP2α antibody. Graphs display averages ± standard deviation from biological replicates. Paired t-test, ****$p < 0.0001$, ns $p = 0.1576$, $n_{\text{all tested genotypes}} = 3$; $t = 47.27$, $df = 3$ (b), $t = 1.874$, $df = 3$ (c).

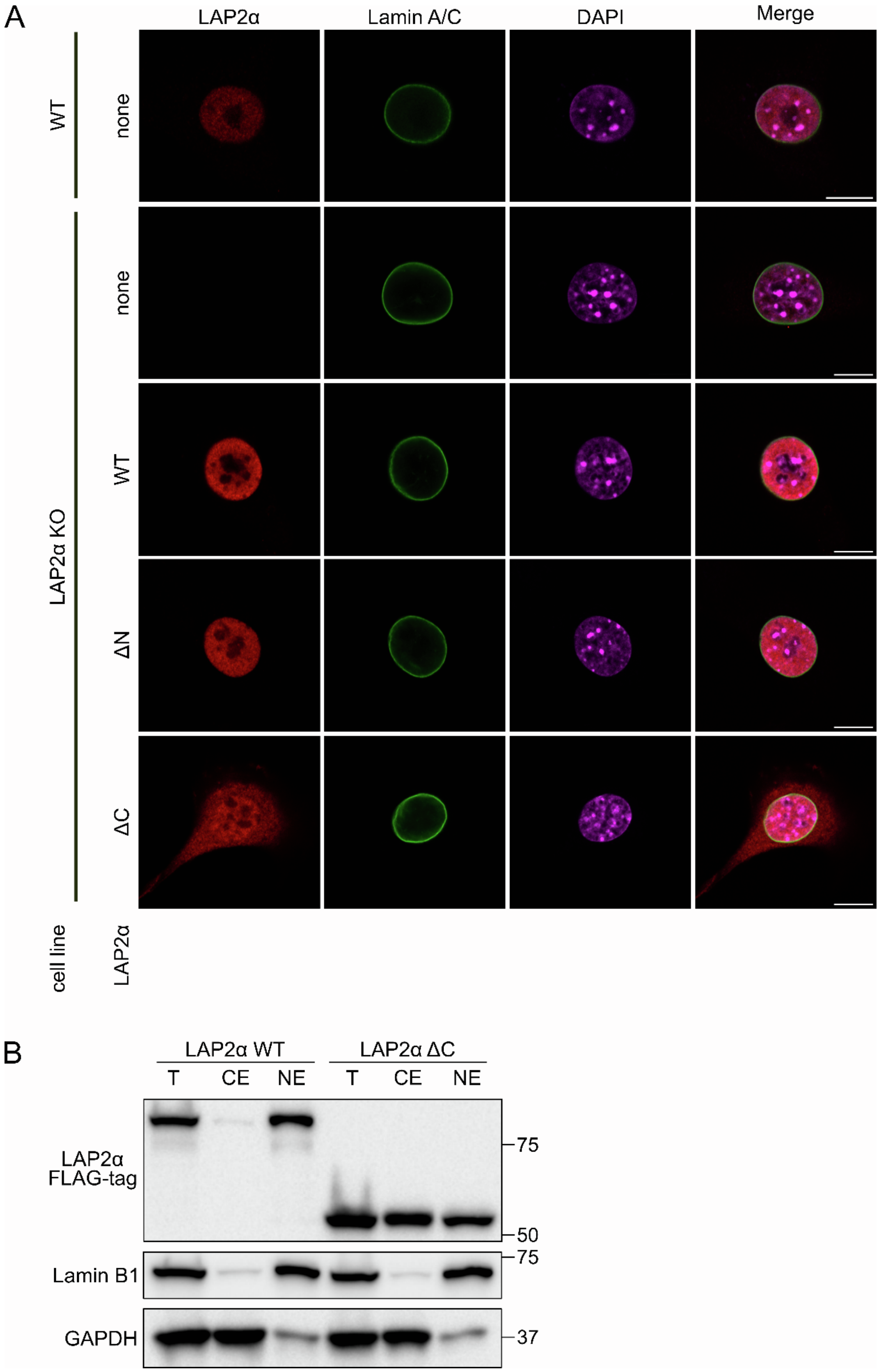

Figure S3. **Cellular localization of endogenous LAP2α and ectopically expressed LAP2α mutants. Related to Figures 3–5. (A)** Immunofluorescence microscopy

images of wildtype (WT) and LAP2α KO cells, and of LAP2α KO cell lines expressing ectopic LAP2α WT, ΔN, and ΔC constructs using LAP2α-specific 245.2 antibody, lamin A/C-specific 3A6 antibody, and DAPI for DNA visualization are shown. Scale bars: 10 μm. **(B)** Nuclear and cytoplasmic fractions of LAP2α KO cell lines ectopically expressing ectopic LAP2α WT and LAP2α ΔC truncation variant were obtained and analyzed by Western blot with the indicated antibodies. T: total; CE: cytoplasmic extract; NE: nuclear extract.

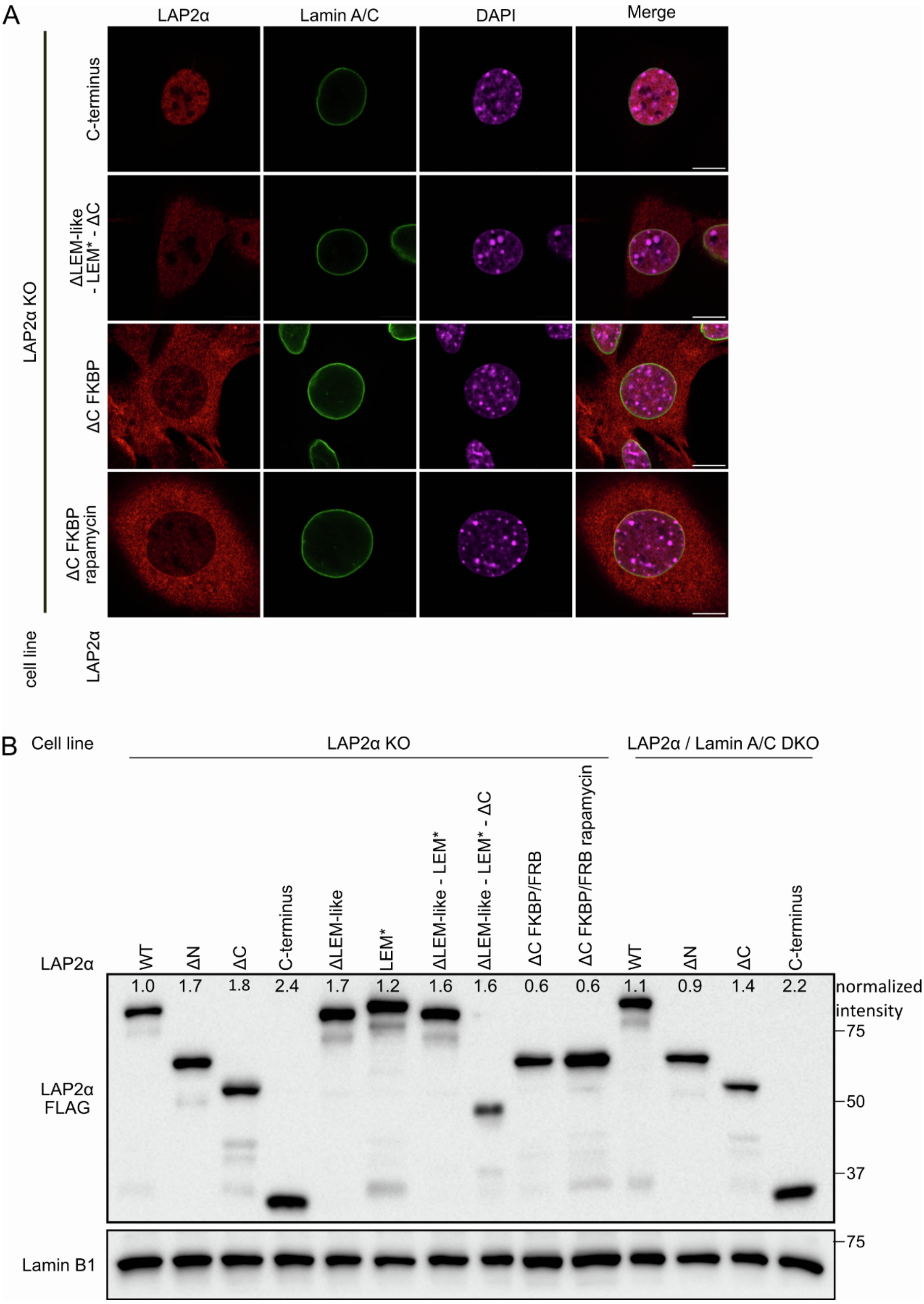

Figure S4. **Cellular localization and nuclear expression levels of ectopic LAP2α and LAP2α mutants. Related to Figures 3–5 and STAR Methods. (A)** Immunofluorescence microscopy images display LAP2α KO cells expressing LAP2α deletion mutants as indicated on the left. ΔC FKBP/FRB: LAP2α KO cells expressing

FLAG-LAP2α-FKBP and HA-LAP2α-FRB, untreated (control) or treated with 1 µM rapamycin for 24 hours. The following antibodies were used: LAP2α-specific 245.2 antibody, lamin A/C-specific 3A6 antibody, and DAPI for DNA visualization. Scale bars: 10 µm. **(B)** LAP2α KO and LAP2α/lamin A/C DKO cell lines ectopically expressing the indicated truncation variants of LAP2α were subjected to nuclear and cytoplasmic fractionation. Nuclear protein extracts were analyzed by Western blot using the indicated antibodies (anti-FLAG, anti-lamin B1). Western blot signals for LAP2α were quantified, normalized to the lamin B1 signal, and are expressed as fold difference to LAP2α KO cells expressing ectopic LAP2α WT.

Table S1. Primers and templates used for PCR to generate fragments for Gibson assembly of indicated vectors. Related to STAR Methods.

Vector	Fragment	Forward primer	Reverse primer	Template
pLVX mCherry -LAP2α WT	FLAG-tag	FP1_Vector_FLAG	RP1_FLAG_Lap2a_ WT	AID 3xFLAG T2A GFP (a kind gift from Christa Buecker)
pLVX mCherry -LAP2α WT	LAP2α WT	FP2_FLAG_Lap2a _WT	RP2_Vector_Lap2a_ WT	cDNA (from LAP2α wildtype mouse dermal fibroblast)
pLVX mCherry -LAP2α ΔN	FLAG-tag	FP1_Vector_FLAG	RP1_FLAG_Lap2a_d N187	AID 3xFLAG T2A GFP
pLVX mCherry -LAP2α ΔN	LAP2α ΔN	FP2_FLAG_Lap2a _dN187	RP2_Vector_Lap2a_ WT	pLVX mCherry- LAP2α WT
pLVX mCherry -LAP2α ΔC	FLAG-tag	FP1_Vector_FLAG	RP1_FLAG_Lap2a_ WT	AID 3xFLAG T2A GFP
pLVX mCherry -LAP2α ΔC	LAP2α ΔC	FP2_FLAG_Lap2a _WT	RP2_Vector_Lap2a_d C279	pLVX mCherry- LAP2α WT
pLVX mCherry -LAP2α ΔLEM- like	FLAG-tag	FP2_FLAG_LEM- like	RP2_Vector_LEM- like	pLVX mCherry- LAP2α WT
pLVX mCherry -LAP2α	LAP2α ΔLEM- like	FP1_Vector_LEM- like	RP1_FLAG_LEM- like	pLVX mCherry- LAP2α WT

ΔLEM like				
pLVX mCherry -LAP2α ΔLEM-like-LEM*	FLAG-tag	FP2_FLAG_LEM-like	RP2_Vector_LEM-like	pLVX mCherry-LAP2α LEM*
pLVX mCherry -LAP2α ΔLEM-like-LEM*	LAP2α ΔLEM-like-LEM*	FP1_Vector_LEM-like	RP1_FLAG_LEM-like	pLVX mCherry-LAP2α LEM*
pLVX mCherry -LAP2α C-term.	FLAG-tag	FLAG_FOR	FLAG_REV	pLVX mCherry LAP2α ΔLEM-like-LEM*
pLVX mCherry -LAP2α C-term.	LAP2α C-term.	C_term_FOR	C_term_REV	pLVX mCherry LAP2α ΔLEM-like-LEM*
pLVX mCherry -LAP2α ΔC-FRB	FLAG-LAP2α ΔC	FP1_Vector_FLAG	RP1_Lap2a_FRB	pLVX mCherry LAP2α ΔC
pLVX mCherry -LAP2α ΔC-FRB	FRB	FP2_Lap2a_FRB	RP2_Vector_FRB	CFP-PCRD-FRB (Addgene #87449)
pLVX puro LAP2α ΔC-FKBP	HA-LAP2α ΔC	FP1_Vector_HA	RP1_Lap2a_FKBP	pLVX mCherry LAP2α ΔC
pLVX puro LAP2α ΔC-FKBP	FKBP	FP2_Lap2a_FKBP	RP2_Vector_FKBP	FKBP-DCRD-YFP (Addgene #87450)

pLVX mCherry -LAP2α ΔLEM-like-LEM*-ΔC	LAP2α ΔLEM-like-LEM*-ΔC	dLEM-like_dC_FOR	dLEM-like_dC_REV	pLVX mCherry LAP2α ΔLEM-like-LEM*
pLVX mCherry -Lamin A WT	FLAG-tag	LMNA.FOR	LMNA.REV	pLVX mCherry-LAP2α WT
pLVX mCherry -Lamin A WT	Lamin A	FLAG.FOR	FLAG.REV	cDNA (from LAP2α wildtype mouse dermal fibroblast)

Primers and templates used for PCR to generate fragments for Gibson assembly of the indicated vectors. The table includes the vector names, corresponding fragments generated, forward and reverse primers used (with sequences provided in Table S3), and the templates from which each fragment was derived.

Table S2: Primers and templates used for Site-Directed Mutagenesis. Related to STAR Methods.

Construct	Introduced mutation	Forward primer	Reverse primer	Template
LAP2α LEM*	GPVV to AAAA	m24_F	m24_R	pLVX mCherry-LAP2α WT
Lamin A S22A	TCG to GCC	S22A.FOR	S22A.REV	pLVX mCherry-Lamin A WT
Lamin A S22D	TCG to GAC	S22D.FOR	S22D.REV	pLVX mCherry-Lamin A WT
Lamin A S22/392A	AGC to GCC	S392D.FOR	S392A.REV	pLVX mCherry-Lamin A S22A
Lamin A S22/392D	AGC to GAC	S392D.FOR	S392D.REV	pLVX mCherry-Lamin A S22D

Primers and templates used for Site-Directed Mutagenesis. The table includes the construct names, introduced mutations, forward and reverse primers used (with sequences listed in Table S3) and the template names from which each construct was derived.

Table S3: List of primer sequences. Related to STAR Methods.

Primer	Sequence (5' - 3')
FP1_Vector_FLAG	AGGATCTATTTCCGGTGAATTCGCCACCATGGACTACAAAGACCATGACGGTGA
RP1_FLAG_Lap2a_WT	CCGGTCTAGAGGGCCCCTTGTCATCGTCATCCTTGTAATCA
FP2_FLAG_Lap2a_WT	GGGCCCTCTAGACCGGAGTTCCTAGAGGACCCT
RP2_Vector_Lap2a_WT	GGGATCCGCGGCCGCTCTAGATTACTGCTTATTTCCACGCTTTTTAATAACTT
RP1_FLAG_Lap2a_dN187	TTCCTCTAGAGGGCCCCTTGTCATCGTCATCCTTGTAATCA
FP2_FLAG_Lap2a_dN187	GGGCCCTCTAGAGGAAAGAAGAAAGAACACAAGAAAGTGAAGT
RP2_Vector_Lap2a_dC279	GGGATCCGCGGCCGCTCTAGATTACTGGCTAAGTCTCTTACATATGTTTCTACA
FP1_Vector_LEM-like	AGGATCTATTTCCGGTGAATTCGCCACCATGGACTACAAAGACCATGACGGT
RP1_FLAG_LEM-like	GCGAGCGGTCTAGAGGGCCCCTTGTCATCGTCATCCTTGTAATCAATATCATGA
FP2_FLAG_LEM-like	CTCTAGACCGCTCGCCGCGGGAGCCAACAG
RP2_Vector_LEM-like	GAGGGAGAGGGGCGGGATCCTTACTGCTTATTTCCACGCTTTTTAATAACTTTGTGTACTTCCCCTCCA
FLAG_FOR	TTTCTTCCATTTCAGGTGTCGTGAGGATCTATTTCCGGTGAATTCGCCACCATGGACTACAAAGACCA
FLAG_REV	TCAGAATCTTGATAGCTGGATCTAGAGGGCCCCTTGTCAT
C_term_FOR	ATGACAAGGGGCCCTCTAGATCCAGCTATCAAGATTCTGAATCCCTG
C_term_REV	TCGGCCAGTAACGTTAGGGGGGGGGAGGGAGAGGGGCGGGATCCTTACTGCTTATTTCCACGCTTTTTAATAACTTTGTG

RP1_Lap2a_FRB	CCAGAGGATCTGGCTAAGTCTCTTACATATGTTTTCTACATCC
FP2_Lap2a_FRB	TTAGCCAGATCCTCTGGCATGAGATGTGG
RP2_Vector_FRB	GAGGGAGAGGGGCGGATCCTCACTTTGAGATTCGTCGGAACACAT
FP1_Vector_HA	CGAGCTCAAGCTTCGAATTCGCCACCATGTACCCCTATGATGTGCCTGACTACGCAGGGCCCTCTAGACCGGAGTTCCTAGAGGA
RP1_Lap2a_FKBP	TGCACTCCCTGGCTAAGTCTCTTACATATGTTTTCTACATCC
FP2_Lap2a_FKBP	TAGCCAGGGAGTGCAGGTGGAAACCAT
RP2_Vector_FKBP	ATTATCTAGAGTCGCGGATCCTTATTCCAGTTTTAGAAGCTCCACATCGAAGAC
dLEM-like_dC_FOR	TTTCTTCCATTTCAGGTGTCGTGAGGATCTATTTCCGGTGAATTCGCCACCATGGACTACAAAGACCA
dLEM-like_dC_REV	TCGGCCAGTAACGTTAGGGGGGGGGAGGGAGAGGGGCGGGATCCTTACTGGCTAAGTCTCTTACATATGTTTTCTACATCC
m24_F	GGCAGCAGGATTCACTCCATATCTTAC
m24_R	GCTGCGGGAACAACCAGGAAGCTATATG
LMNA.FOR	CCTCTAGAGAGACCCCGTCACAGCG
LMNA.REV	AGGGGCGGGATCCGCGGCCGCTTACATGATGCTGCAGTTCTGGGAG
FLAG.FOR	GAGGATCTATTTCCGGTGAATTCGCCACCATGGACTACAAAGACCA
FLAG.REV	GGGGTCTCTCTAGAGGGCCCCTTGTCATCG
S22D.FOR	TACCCCACTGGACCCCACTCGGATCACCCGG
S22D.REV	GAGCTGGCCTGCGCCCA
S392D.FOR	CCTGTCCCCCGACCCTACCTCGC
S392D.REV	CGCAGCCTCTCCTCCTCG
S22A.FOR	TACCCCACTGGCCCCCACTCGGATCAC

S22A.REV	GAGCTGGCCTGCGCCCCA
S392A.FOR	CCTGTCCCCCGCCCCTACCTCGC
S392A.REV	CGCAGCCTCTCCTCCTCG

List of primer sequences used in this study, including the names of the primers and their corresponding sequences.

Supplementary Reference

[S1] Gesson, K., Rescheneder, P., Skoruppa, M.P., Von Haeseler, A., Dechat, T., and Foisner, R. (2016). A-type Lamins bind both hetero- and euchromatin, the latter being regulated by lamina-associated polypeptide 2 alpha. Genome Research *26*, 462-473. 10.1101/gr.196220.115.
